# The Great Healing Potential Hidden in Plant Preparations of Antioxidant Properties: A Return to Nature?

**DOI:** 10.1155/2020/8163868

**Published:** 2020-10-09

**Authors:** Małgorzata Kiełczykowska, Irena Musik

**Affiliations:** Chair and Department of Medical Chemistry, Medical University of Lublin, 4A Chodźki Street, 20-093 Lublin, Poland

## Abstract

The application of chemicals in industry and agriculture has contributed to environmental pollution and exposure of living organisms to harmful factors. The development of new pharmaceutical agents enabled successful therapy of various diseases, but their administration may be connected with side effects. Oxidative stress has been found to be involved into etiology of numerous diseases as well as harmful action of drugs and chemicals. For some time, plant origin substances have been studied as potential protective agents alleviating toxicity of various substances and symptoms of diseases. The aim of the current review was to present the diversity of the research performed during the last five years on animal models. The outcomes showed a huge protective potential inherent in plant preparations, including alleviating prooxidative processes, strengthening antioxidant defence, ameliorating immune parameters, and reversing histopathological changes. In many cases, plant origin substances were proved to be comparable or even better than standard drugs. Such findings let us suggest that in the future the plant preparations could make adjuvants or a replacement for pharmaceutical agents. However, the detailed research regarding dose and way of administration as well as the *per se* effects needs to be performed. In many studies, the last issue was not studied, and in some cases, the deleterious effects have been observed.

## 1. Introduction

In the recent centuries, a great change of the conditions of the human life has taken place, due to the development of industry, agriculture, medicine, and pharmacy. The new synthetic substances were applied to protection of crops as well as successful therapy of various diseases. However, except for beneficial influence, consisting in numerous facilitations of human existence and extending human life span, negative effects have also occurred [1, 2]. A growing pollution of natural environment, resulting from the industry development, has been observed for many years [3–5]. Despite the efforts aiming at alleviating and preventing this phenomenon, it still belongs to the most important problems to be solved by the mankind. The application of plant protection products has made another contribution to environmental contamination [6–9]. As it is not possible to improve the situation immediately, many people, i.e., industrial workers or farmers are still exposed to harmful substances like heavy metals [4, 10–12], organic chemicals (e.g., CCl_4_) [13, 14], or pesticides and plant growth regulators [8, 15]. Another problem results from introducing plenty of new pharmaceutical agents. They allowed to relieve suffering of many subject and successive treatment in cases of mortal diseases. However, on the other hand, plenty of side effects have also been observed [16, 17]. Analgesic and antipyretic drugs like acetaminophen or aspirin can induce liver and kidney damage [18–22]. The antineoplastic agents can cause severe disturbances like testicular damage [23], nephrotoxic [16, 24], cardiotoxic [25, 26], and hepatotoxic [27] effects. Antibiotics have also proved to show side effects including liver and kidney damage [28, 29]. The widespread practice of using different additives to preserve and improve the taste of food makes another source of toxic action on human organisms [30]. The growing life span is connected with an increase in incidence of neurodegenerative disorders like Parkinson' disease [31] or Alzheimer's disease [32]. Furthermore, in the recent decades, obesity, hyperlipidemia, and connected disturbances have become a significant worldwide problem [33, 34].

All the presented facts have made the scientists search for any agents which could exert protective effects against toxic environmental pollutants and chemicals used in industry and agriculture and replace the standard drugs or make beneficial adjuvants. The return to natural products, sometimes used for thousands years in traditional medicine, has become one of the significant directions of this research [35–39]. It could be stated, without any exaggeration, that “a great return to nature” is recently being observed. Plant extracts and plant origin substances *per se* do not show so many side effects as pharmacological substances. On the other hand, they contain numerous compounds of anti-inflammatory and antioxidative properties like polyphenolic derivatives and flavonoids [40–42]. The presented facts prompted large-scale research on possibilities of application of plant preparations as protective agents against toxicity of various substances as well as adjuvants alleviating the symptoms of diseases, obesity, and traumata [6, 25, 38, 43–49]. A great quantity of plant species have been investigated, and the obtained results seem to be very promising. Some studies have included the comparison of the investigated materials with standard drugs, and the outcomes suggest that in many cases the replacement would be possible [20, 31, 35, 50–53]. However, many questions remain to be solved as to the best way of treatment and the most beneficial dose.

Oxidative stress—the disturbed balance between generation of reactive oxygen species (ROS) and antioxidants' level in an organism—has been found to be involved, less or more, into the etiology of most diseases [54–56]. This process involves the generation of ROS, active particles capable of injuring all bioactive compounds—protein, lipids, and nucleic acids in an organism. Lipid peroxidation caused by ROS may lead to damage of membrane lipids. Living organisms developed a wide range of endogenous substances, both enzymatic and low-molecular ones, which can neutralize ROS. Negative effects resulting from stress, exposure to toxic substances, side effects of the standard drugs, or even food supplements have also been proved connected with prooxidative processes and deterioration of antioxidant defence [13, 30, 40, 57, 58]. Plant origin preparations, in turn, have been found to exert a strong antioxidant action due to high content of components of antioxidative properties. Numerous studies have revealed their direct influence on oxidative processes by reducing lipid peroxidation or protein carbonylation as well as increase in antioxidant enzymes' activities and low-molecular antioxidant concentrations [34, 43, 59, 60].

Different pathways involved in oxidative and inflammatory processes have been found to be affected in the course of protective action of plant preparations.

The studies have shown the involvement of Nrf2 and Keap1 proteins. Nrf2 is regarded as a key transcription factor mediating the endogenous antioxidant response, and Keap1 is its negative regulator. Under oxidative stress conditions, Nrf2 is released and translocated to the nucleus where it binds to ARE regions in DNA and stimulates antioxidant enzyme gene expression. Both Nrf2 and Keap1 as well proteins regulated by Nrf2, responsible for defence against antioxidant stress like HO-1 and *γ*-GCS, have been found to be disturbed by harmful factors (cadmium or high-fat diet) and regulated by plant preparations [12, 61]. Plant origin substances have been reported to cause upregulation of Nrf2, HO-1, and *γ*-GCS in both nonexposed and Pb-exposed rats [62].

Another pathway connected with oxidative and inflammatory processes which have been proved involved into protective properties of plant preparations is NF-*κ*B pathway. NF-*κ*B is a transcription factor responsible for expression of proinflammatory cytokines [56]. Its activation can be triggered by TLR receptors, belonging to pattern recognition receptors. The inflammation and redox balance have been found to be strongly connected with each other [63]. Interleukins, *γ*-interferon and tumour necrosis factor, have been found to affect ROS production [64]. The involvement of the LPS-TLR4-NF-*κ*B pathway into protective action of plant materials has been reported [56, 65]. Other authors have stated that a protective effect of a plant extract, resulting from antioxidative and anti-inflammatory influence observed in diabetes animal model, could be attributed to inhibition of NF-*κ*B activation [66].

Mitogen-activated protein kinases (MAPK) belong to enzymes which make mediators of various processes occurring in cells like death, proliferation, or differentiation. The MAPK pathway begins from a signal from an extracellular receptor and through a cascade of subsequent protein phosphorylation leads to activation of different proteins including transcription factors (e.g., p53 protein) and finally to the expression of genes. ROS have been proven to be connected with particular steps of the MAPK pathway. There are three kinds of MAPK in mammals: p38 MAPK, ERK1 (extracellular signal-regulated kinase 1), and JNK (c-Jun N-terminal kinase) [64, 67]. The studies on plant revealed the influence of the studied plant materials on some elements of the MAPK pathway [42].

The next pathway connected with oxidative stress which has been found affected by plant preparations is the JAK/STAT pathway. An outside signal, usually being a cytokine, binds to a membrane receptor resulting in its dimerization. The next stage is the activation of JAK which renders possible phosphorylation of receptor, which in turn enables STAT binding and phosphorylation. Activated by phosphorylation STAT is then translocated into the nucleus where it acts as a transcription factor [68]. The relationships between this pathway and ROS have been reported [69]. In the current study, disturbances of oxidative balance as well as bone marrow damage and reduction in pJAK2/JAK2 and pSTAT5a/STAT5a, caused by radiation exposure, have been found to be improved by a plant preparation, which made the authors suggest that the studied material can stimulate the JAK2/STAT5a signal pathway [70].

The aim of the current review is to present the results of the studies performed in the last five years regarding the protective and medicinal properties of plant origin preparations with particular emphasis on their antioxidant action.

## 2. The Protective Properties of Plant Preparations against Toxicity of Various Factors

### 2.1. The Protective Influence of Plant Preparations against Chemicals Applied in Agriculture

The use of different chemicals in food production has been growing dramatically in the recent years, causing the contamination of the natural environment and increasing thread to human health. Neurotoxicity, hepatotoxicity, reproductive disturbances, and cancerogenesis belong to the negative effects exerted on organisms [6, 8, 15, 71]. Moreover, chemicals of lipophilic character can be accumulated in membranes [9]. Enhanced generation of ROS was proved involved into their harmful influence [6, 9, 71]. Plant origin materials were revealed to show protective properties not only by strengthening the antioxidant barrier but also by reversing histopathological changes. A wide range of different materials was studied, including simple extracts [2, 6, 7] as well as commercial products [9, 15]. The saponins from *Tribulus terrestris*, which were reported to possess antiaging action, were found to exert protective effects against rotenone-induced parkinsonism [15].

The details concerning the above mentioned studies are presented in Table 1.

### 2.2. The Protective Influence of Plant Preparations against Toxic Effects of Heavy Metals

Environmental pollution with heavy metals makes a great global problem. The most toxic ones are lead, cadmium, and mercury. Even low concentrations can cause severe disturbances of organism, including brain, hepatic, renal, and reproductive damage [10]. As their harmful action is connected with oxidative stress induction, plant extracts showing antioxidant properties were studied as possible protective agents and the obtained results were found to be promising [10, 11], although the accumulation of a toxic metal could not be prevented in every case [62].

The ability of plant origin substances proanthocyanidins to prevent lead-induced hepatotoxicity was suggested to be connected with the Nrf2/ARE pathway (as an increase of mRNA expression levels of Nrf2 in the liver of mice administrated with proanthocyanidins and/or lead was observed) as well as with the reduction of endoplasmatic reticulum stress *via* a decrease in stress-related proteins GRP78 and CHOP [62].

In the experiments concerning the toxicity of mercury, plant extracts relieved the negative effects in the case of both an inorganic form (mercury (II) chloride) and organic one (dimethylmercury); at the same time, the beneficial influence included not only oxidant and immunological parameters but also histopathological changes [4, 72, 73].

Plant extracts were also found to exert wide protective effects against the third most dangerous heavy metal cadmium, with the results being confirmed by *in vitro* studies with using murine hepatocytes [3]. Additionally, an animal study showed the involvement of the Nrf2/Keap1 pathway into the protective action of *Pyrantha fortuneana* extract as the plant material, given both alone and coadministered with cadmium caused a significant increase in expression of Nrf2 and decrease in expression of Keap1 in the kidneys of rats vs. the control and Cd-exposed group, respectively [12].

Apart from lead, mercury, and cadmium the research concerning metals' toxicity included also liver injury caused by iron overload. 70% methanol extract of *Drosera burmannii* Vahl. showed a distinct, dose-dependent efficacy against iron-induced hepatoxicity. This effect, particularly in case of the highest dose, was comparable with that exerted by a standard drug desirox—an iron chelator. Additionally, the studied extract studied *in vitro* showed the ability to chelate Fe^2+^ ions. Such findings made the authors suggest that the studied preparation might be used as a medicine in cure of iron overload-induced diseases [50].

The details concerning the above-mentioned studies are presented in Table 2.

### 2.3. The Protective Influence of Plant Preparations against Various Chemicals

Plant origin substances, extracts and their particular fractions, essential oils, and seed powders were found to reverse or alleviate disturbances of organism resulting from exposure to different chemicals. The resent research included various compounds, e.g., hepatotoxic tetrachloromethane (CCl_4_) used for years as a solvent and now regarded as an environmental pollutant [14], aluminium known for its neurotoxicity [74], aflatoxins produced by toxigenic fungi which made food contaminants [75], and chemicals used in industry and laboratory experiments like thioacetamide [76] or 1-chloro-2,4-dinitrobenzene [77]. Additionally, plant origin substances were proved to decrease mouse mortality resulting from the acute CCl_4_ toxicity [78] as well as from Concanavalin A exposure [79]. Furthermore, several studies included the influence of plant materials alone, and generally, no harmful effects were observed [14, 54, 74, 75, 78, 80, 81].

The details of the performed studies are presented in Table 3.

### 2.4. The Protective Influence of Plant Preparations against Carcinogens

Plant extracts were studied using animal model as for their possible application in tumour therapy due to the presence of anticancer and antioxidant components. The necessity of searching for new agents, suitable for tumour treatment, was challenged by the lack of effective chemotherapy and severe side effects associated with the used medicines. The obtained results seem to be promising as the studied materials alleviated carcinogen-induced oxidative stress as well as disturbances of immunological parameters [47, 88, 89]. Histolopathological studies confirmed the beneficial effects of the investigated preparations [46, 89].

The detailed results of the performed studies are presented in Table 4.

### 2.5. The Protective Influence of Plant Preparations against Ethanol

Alcohol excessive consumption and addiction leads to different negative effects: liver injuries including steatosis [56, 91], brain damage [92], and reproductive system damage [93]. The disturbances of oxidative balance were suggested to be one of the factors underlying alcohol toxicity, which was confirmed by intensification of lipid peroxidation as well as deterioration of antioxidant barrier, observed in animals exposed to ethanol [44, 56, 91]. The involvement of oxidant stress into ethanol toxicity was also showed by Phunchago et al. [92] who observed the protective influence of antioxidant vitamin C. The studies presented in the current review proved that plant materials showed a wide range of protective actions including ameliorating of liver markers, oxidative parameters, and alleviation of histopathological changes [56, 91, 92]. The involvement of LPS-TRL4-NF-*κ*B pathway was also been proved [56, 65].

The detailed results of the performed studies are presented in Table 5.

### 2.6. The Protective Influence of Plant Preparations against Toxic Effects of Antipyretic and Analgesic Drugs

The application of antipyretic and analgesic drugs has been growing rapidly in recent years. Acetaminophen, known also as paracetamol, N-acetyl-p-aminophenol or APAP, a substitute of aspirin, is one of the most often used over-the-counter medicines. However, its application can cause severe hepatotoxicity, and this fact is all the more dangerous because of possible overdose resulting from self-administration [18, 22, 40, 95]. Another side effect, connected with paracetamol application, is nephrotoxicity [22, 40]. The harmful action of acetaminophen includes oxidative stress, particularly the depletion of GSH and protein sulfhydryl groups blocking [96]. Several studies revealed the possibility of plant extract application as protective adjuvants, wherein *Moringa peregrine*, *Genista quadriflora*, *Teucrium polium geyrii*, and *Cassia surattensis* showed the best influence which included not only amelioration of liver damage markers but also improvement of antioxidant parameters and reduction of the lipid peroxidation process [19, 20, 96].

The details concerning the mentioned investigations are presented in Table 6.

### 2.7. The Protective Influence of Plant Preparations against Toxic Effects of Antibiotics

Not only are antibiotics used for the treatment of Gram-negative bacterial infection, but they also can cause side effects to occur [98, 99]. Gentamycin belongs to those which are used in case of strains resistant to other antibiotics, but its application can lead to hepato and nephrotoxicity [28]. The similar properties are shown by an antibiotic polymyxin, whose application was ceased because of its nephrotoxicity, but an increased drug resistance of Gram-negative strains has rendered it being used again [99]. Oxidative stress as well as inflammation processes was suggested to take part in the development of the mentioned negative effects [29, 98, 99]. Materials obtained from plants containing antioxidant components and affecting immune functions were investigated as to their possible protecting application, and the results seem to be promising although they also pointed to the necessity of taking proper precautions and precise choosing the dose as in some cases the higher dose showed a better influence [29, 98], while other authors reported quite opposite results [28].

The details concerning the above mentioned issues are presented in Table 7.

### 2.8. The Protective Influence of Plant Preparations against Toxic Effects of Anticancer Drugs

Cancer successful therapy is often a great problem because of the side effects of the applied agents which include deterioration of reproductive proficiency [23], nephrotoxicity [16, 100], and hepatotoxicity [37] and cardiotoxicity [26]. The results of the studies presented below clearly show that the toxic action of anticancer drugs is strongly connected with the prooxidative processes, deterioration of antioxidant barrier, and histopathological changes. The extracts of different plants, used as spices and drugs in traditional medicine for centuries, were studied for their protective potential [101]. Both simple extracts and particular fractions [25, 26] proved their protective properties which included the amelioration of the disturbed oxidant parameters. Antioxidant and antiapoptotic properties of plant extracts were confirmed by *in vitro* investigations performed on renal tubular epithelia cells [100] and cardiomyocytes [25]. Histopathological changes were also found to be relieved by plant materials [16, 23, 24, 26, 102].

The details concerning the above mentioned issues are presented in Table 8.

### 2.9. The Protective Influence of Plant Preparations against Side Effects of Various Drugs

Many drugs' administration is accompanied with numerous side effects which cause life complication and may negatively influence patients' compliance. These facts prompted the searching for any adjuvants reversing or at least alleviating side effects. Recently, a growing interest in plant origin substances, often those used in traditional medicine, is being observed [104, 105]. The studies presented below revealed the effectiveness of plant extracts against toxicity of diverse drugs: psychiatric (lithium carbonate), thyrostatic (Propylthiouracil), and cardiac activity stimulators (Isoproterenol), a contrast medium (Iodixanol), and dermatological medicine (Triamcinolone acetonide). The negative influence of the studied drugs often included deterioration of antioxidant barrier [105, 106]. The studied preparations showed beneficial effects, and *in vitro* studies confirmed their antioxidant potential [104, 107].

The details concerning the above mentioned issues are presented in Table 9.

### 2.10. The Protective Effects of Plant Preparations Observed in Animal Models of Different Disorders

#### 2.10.1. The Protective Effects of Plant Preparations Observed in Arthritis

Arthritis has recently become a serious, worldwide problem as this disease is related with pain and physical disability, and no effective therapy except for a surgery can be applied [49]. As the risk of it increases with age, the growing spam life contributes to the still enhancing incidence. Plant substances were found to prevent enhancement of the proinflammatory cytokines like IL-1*β*, IL-6, and TNF-*α*, involved into osteoarthritis pathogenesis of OA. Additionally, the reduction of metalloproteinases responsible for joint damage as well as upregulation of their inhibitors—TIMPs and extracellular matrix components—was observed [48, 49]. Furthermore, plant origin substances were reported to reverse oxidative parameters' disturbances, also taking part in osteoarthritis development [55].

The detailed outcomes of the performed studies are collected in Table 10.

#### 2.10.2. The Protective Effects of Plant Origin Materials in Cases of Neurodegenerative Disorders

The next type of disorders studied with using an animal model was neurodegenerative diseases. ROS have been reported to be involved in the development of Alzheimer's, Huntington's, and Parkinson's diseases. Extracts obtained from plants used in traditional Chinese and Ayurveda medicine, possessing numerous therapeutic properties and containing antioxidant components, were shown to exert a considerable beneficial influence [31, 32, 39].

Traumatic brain injury, regarded as a worldwide grave challenge being a cause of many death and disability cases, is considered to need an effective therapy. Water extracts of plant commercial products were revealed to show a beneficial influence on immunological and oxidative parameters [38, 60].

Psychiatric disorders like anxiety or depression were also proved to be connected with neurodegenerative disturbances as well as changes of immunological and oxidative parameters which were alleviated by plant extracts [111, 112].

The detailed outcomes of the performed studies are collected in Table 11.

#### 2.10.3. The Protective Effects of Plant Origin Materials in Cases of Animal Menopause Model

In menopausal women, the deficiency of sex hormones can lead to various disturbances of organism. The research concerning hormone replacement therapy showed that it can cause different side effects, so the attention was paid to nonpharmaceutical agents, all the more because plant flavonoids were proved to possess phytoestrogen properties [114]. In the performed studies, plant origin materials improved some elements of lipid profile and oxidative parameters deteriorated by ovariectomy [115]. Bone mineral density in rats was also ameliorated, although this effect became less distinct along with lengthening of the experiment [114]. However, in some cases, the obtained results were not so distinctly beneficial [116].

The details of the performed studies are collected in Table 12.

#### 2.10.4. The Protective Effects of Plant Origin Materials in Lung Disorders

Plant origin substances were shown to possess some efficacy against lung disorders, and the beneficial effect included the improvement of oxidative and inflammatory parameters, morphological disturbances, and factors controlling extracellular matrix functions and vascular homeostasis [117–119].

The details of the performed studies are collected in Table 13.

#### 2.10.5. The Protective Effects of Plant Origin Materials in Lipid Profile Disturbances

Lipid profile disturbance is associated with severe diseases like diabetes as well as hepatic and cardiovascular disorders [120]. It is rated among the main factors causing disability and death [34]. Factors which were used to induce such a condition were also found to cause intensification of prooxidative processes connected with deterioration of antioxidant defence and DNA damage. Plant preparations proved to display beneficial effects, although only in one case *per se* influence was studied [120].

The detailed results of the performed studies are collected in Table 14.

#### 2.10.6. The Protective Effects of Plant Origin Materials in Ischaemia/Reperfusion Model

Ischaemia/reperfusion damage is a serious problem which can occur as a consequence of surgery, e.g., transplantation or coronary bypass, and may lead to severe injuries, resulting among other things from increase in ROS generation. Pretreatment with plant materials, obtained from species used in traditional medicine, was found to be effective against prooxidative processes and histopathological changes observed in ischaemia/reperfusion animal model [123–126].

The detailed results of the performed studies are collected in Table 15.

#### 2.10.7. The Protective Effects of Plant Origin Materials in Animal Model Diabetes

Plant origin substances were observed to be effective at reversing disturbances observed in the course of diabetes. A wide range of agents was studied, simple extracts, combinations of two extracts as well as an oil, banana pasta or substances separated from plant material. A great variety of species was investigated, including herbs, fruits, or vegetables. Many of them had been known as being useful in different fields of medicine, sometimes from ancient times [66, 127–129]. In several studies, biochemical, oxidant, and inflammatory parameters in the blood and organs including the lens, brain, liver, pancreas, kidney, and heart were found considerably improved or restored by different materials [42, 57, 66, 128, 129]. Histopathological investigation revealed that plant preparations showed a considerable ability to attenuate pancreas, kidney, and liver damage observed in the animal model of diabetes [127, 128, 130]. The beneficial influence of plant extracts on the deterioration of sperm quality [131], diabetes-associated cataract, and retinopathy and an ability to suppress MAPK signal transduction [42] and the amyloidogenic pathway [57] were reported. However, there are limitations of these investigations as in most of them the effect of the applied plant substance was not studied.

The detailed results of the performed studies are collected in Table 16.

#### 2.10.8. The Protective Effects of Plant Origin Materials in Animal Model of Obesity

Plant origin preparations were also investigated as to their potential to prevent pathological processes connected with obesity. This direction seems to be of great importance as obesity and related disturbances, resulting from sedentary lifestyle as well as excessive consumption, are becoming more and more serious world problem [132, 133]. Diet-induced obesity was found to be connected with different disturbances of various parameters including liver markers and lipid profile as well as inflammation, lipogenesis, and oxidative balance. The performed research revealed the protective effect of plant preparations which involved improvement of oxidative balance by activation of the Nrf2/HO-1 antioxidant pathway [61] and the ameliorating effect on lipid status [132, 133]. The latter was also confirmed *in vitro* as the lipolysis-promoting influence in 3T3-L1 cells was observed [61]. In mice fed a high-fat diet, coadministration of *Euterpe oleracea* extract reversed the changes of lipogenic proteins expression in the liver. Additionally, the investigated preparation increased hepatic expression of ABCG5 and ABCG8 transporters responsible for removing excess of cholesterol by secretion into bile [43].

The detailed results of the performed studies are collected in Table 17.

#### 2.10.9. The Protective Influence of Plant Preparations against Gastric Ulcer-Inducing Factors

The gastric ulcers are regarded as the most common disease of gastrointestinal tract, but they can be caused by many various factors. Therapy of different disorders with using nonsteroidal anti-inflammatory drugs like indomethacin and excessive consumption of alcohol belong to important ones [45, 134]. As pharmaceutical agents are not fully effective and their application may also be connected with development of side effects, an alternative ways of treatment became the subject of research. Plant origin substances showed a wide range of beneficial effects, including alleviated or reversed oxidative stress which was found to be involved into gastric ulcer etiology as well as mechanism of indomethacin toxicity [135–137].

The detailed results of the performed studies are presented in Table 18.

#### 2.10.10. The Protective Effects of Plant Origin Materials in Animal Model of Different Disorders

The symptoms of many other various disturbances were affected in a positive way by using plant materials of experimentally confirmed antioxidant properties and containing acknowledged antioxidants like phenolic acids and flavonoids [36, 41, 138]. A wide range of diseases: lithiasis, thyroid disorders, retinal degeneration, irritable bowel syndrome, hyperthyroidism, periodontitis, and mammary gland hyperplasia, were studied, pointing to great possibilities “hidden” in plant origin agents.

The details of the performed studies are presented in Table 19.

### 2.11. The Protective Effects of Plant Origin Materials against Radiation

Plant extracts also showed some efficacy against radiation-induced damage. The research revealed the involvement of JAK-STAT pathway into the mechanism of the protective influence on the hematopoietic system [70]. Animal studies also showed the reversing effect of plant material on the disturbances of antioxidant defence caused by *γ*-radiation in blood and organs [145, 146]. One of the recent *in vitro* investigations, performed on keratinocyte cells, confirmed the antioxidant action of a plant preparation against UVB radiation, which could contribute to development of new skin protective strategies [147].

The details of the performed studies are presented in Table 20.

## 3. The Comparison of Plant Preparations with Standard Drugs and Supplements: The Dependence of Effects on Treatment Way and Doses

The effects of plant origin materials were often investigated in comparison with those shown by standard drugs. The results clearly show that there may be a possibility to replace different medicines with plant preparations which do not cause so many severe side effects, but the detailed research must be performed before the final conclusions can be made.

The question of advantage of plant origin extracts over any standard drug is complicated, and a univocal answer is very difficult as in some cases the differences in the influence of the compared agents were strongly dependent on the applied dose [45, 135] and studied parameters [115, 129].

Hamm et al. [116] found that hops (*Humulus lupus* L.) flavonoid-rich extract protected against ovariectomy-induced visceral adiposity and increase in liver triglycerides observed in 7-month-old retired breeder C57BL/6 mice. However, particularly in case of the former parameter, the observed effect was not so distinct as that noted in animals receiving 17-*β*-estradiol. Additionally, hops extract could not prevent the loss of uterus weight caused by the surgery while estrogen showed quite a considerable reversing effect.

Leung et al. compared the effect of black tea extract with that exerted by estrogen in ovariectomized rats. The results were characterized by a considerable diversity. In case of body weight reduction, estradiol's benefit influence was much better. In contrast, aortic cGMP and serum TC and peNOS, NOX2, NOX4, and ROS generation in the aorta were affected in similar or even entirely the same way by both agents. Additionally, in case of serum TG the plant materials proved to possess much better ability to restore the values observed in sham-operated control animals [115].

### 3.1. The Comparison of Crude Extracts and the Particular Substances Separated from Plant Materials

The plant extract can show better properties than particular compounds administered alone as there may occur some synergistic effects among its components. Such an assumption could be supported by the results reported by He et al. [148] who compared the influence of *Paeonia suffruticosa* seed extract and 10 compounds (oligostilbenes) isolated from it with using an *in vitro* IL-1*β*-induced osteoarthritis model. In the study performed on rabbit chondrocytes, IL-1*β* caused a significant decrease in viability and 10 studied components used alone showed an improving effect, but the intensity of this effect was markedly different, depending on the structure. However, the application of the extract containing all the oligostilbenes showed the best effect, comparable with that observed for diacerein—a drug of IL-1*β*-inhibition action, used in osteoarthritis therapy. Such results made the authors suggest the possibility of synergism among the particular oligostilbenes.

### 3.2. Plant Preparations as Lipid Profile Regulating Agents – The Comparison with Standard Drugs

Plan preparations were studied in regard to the possibility of their application as lipid profile normalizing agents, and the results seem to be auspicious [120–122]. Additionally, the researchers performed comparisons with standard drugs. The outcomes showed that the efficacy of plant material needs not be worse than that showed by acknowledged pharmaceutical agents. However, it should be emphasized that the final effects may be different, depending on the applied dose.

Veber et al. [34] compared the effects of two doses (125 or 250 mg/kg) of aqueous extract of red cabbage with that showed by fenofibrate, a drug used in therapy of abnormal lipids in the blood, on oxidative stress parameters in rats with hyperlipidemia caused by Triton WR-1339. Although the higher dose proved to possess protective properties generally comparable with the drug, the lower one in some cases showed no efficiency.

*Erica multiflora* L. leaf methanolic extract (150 or 250 mg/kg) effect was studied in rats with Triton WR-1339-induced hyperlipidemia in comparison with fenofibrate taking into account chosen lipid and antioxidant parameters. The higher dose showed comparable or even better protective action than the medicine. In contrast, total DNA damage was alleviated much better by the used drug [120].

Onyenibe et al. [122] investigated the efficiency of two doses of *Monodora myristica* aqueous extract as a protective agent in case of deterioration of lipid profile and oxidative parameters in hypercholesterolemic rats and compared the obtained results with those noted for a standard drug Questran. The authors found that generally two doses of plant preparation, namely, 100 and 200 mg/kg b.w., showed a better influence.

The effects of lipid-lowering drugs simvastatin and ciprofibrate as well as aqueous extract of *Campomanesia adamantium* O. Berg root were compared in rats with high-fructose diet-induced hyperlipidemia. The plant material exerted the entirely comparable influence in case of lipid parameters and even better as regards body weight gain lowering [121].

### 3.3. The Comparison of Plant Extracts of Hepatoprotective Properties with Pharmaceutical Agents

Liu et al. [51] compared the protective action of pretreatment with *Sonneratia apetala* fruit water extract (100, 200, and 400 mg/kg) against hepatic damage caused by acetaminophen in mice with the effect of an acknowledged antidote NAC (N-acetyl-L-cysteine). The scientists observed the protective influence of plant preparation, which was not only comparable but in some cases better—particularly for serum ALT and AST as well as hepatic lipid peroxidation, antioxidant enzymes, TNF-*α*, and IL-6.

Similarly, the rich polyphenol fractions of methanolic extracts of *Genista quadriflora* Munby and *Teucrium polium geyrii* Maire showed a protective effect against hepatoxicity of acetaminophen (APAP) and the comparison with *N*-acetylcysteine showed their comparable properties with the drug, except for histological changes where *Teucrium polium geyrii* Maire exerted a better influence than two other studied agents [20].

### 3.4. The Comparison of Plant Extracts with Silymarin, an Acknowledged Dietary Supplement of Hepatoprotective Properties

Many drugs and chemicals are distinguished by their hepatoxic effects. In view of the increasing environmental pollution as well as more and more common application of different drugs, often over-the-counter ones, the protective agents are highly desirable [18]. Silymarin, a preparation obtained from *Silybum marianum* L., has been used as a hepatoprotective adjuvant for years. Currently, the attention has been pointed to other plants, often those used since antiquity in traditional medicine [44]. Different hepatotoxicity animal models were used to perform comparison with silymarin.

The possibility of replacing silymarin by an extract of *Cassia fistula* L. leaves was studied by Kaur et al. [35] in rats exposed to thioacetamide. The studied extract was applied at three doses (50, 100, and 200 mg/kg b.w.), and beneficial effects of the two higher ones were not worse than those observed in rats receiving silymarin.

The similar observations were reported by Fahmi et al. [53] who compared the protective properties of dietary ginger against diethylnitrosamine hepatotoxicity in rats with those showed by silymarin and found that the studied material in the form of ginger powder or essential oil exerted the same or even better beneficial action.

Choi et al. [46] studied the possibility of *Centella asiatica* leaf ethanol extract (100 or 200 mg/kg) using as a protective agent against dimethylnitrosamine-induced hepatotoxicity in rats. The authors determined different inflammatory cytokines and mediators, liver injury markers, oxidant parameters, and histophological changes. In some cases (liver histology, serum IL-1*β*, TNF-*α*, and IL-2), both doses of the extract showed a better action than silymarin. In case of AST, ALP, IL-6, and INF-*γ* or liver MDA, both agents displayed the similarly significant properties. Furthermore, liver antioxidant enzymes were best ameliorated by the higher dose of plant preparation.

El-Hadary and Ramadan [52] in turn stated that *Moringa oleifera* leaf extract displayed protective properties against hepatotoxic action of diclofenac sodium. The comparison with silymarin proved that the plant preparation exerted comparable or even better effect.

Ahmad and Zeb [18] in turn compared an effectiveness of silymarin and different doses of water extract of *Trifolium repens* leaves against acetaminophen-induced hepatoxicity in mice. The authors reported that in case of most studied hematological, serum biochemical, and liver oxidative parameters, the effect of the highest dose was not worse than that shown by silymarin.

However, the comparison of protective action of leaf extract of *Solanum surattense* with that observed for silymarin in CCl_4_-exposed rats revealed that the latter had entirely better effect regarding liver injury markers and lipid profile in serum as well as lipid peroxidation process in the liver [83].

On the other hand, Dogan and Anuk [44] observed that in ethanol-exposed rats water extract of leaves of *Platantus orientalis* L. generally showed the protective effects comparable or even better than those observed in silymarin-given animals. Interestingly, in case of some parameters, both silymarin and extracts showed insufficient effectiveness.

The possibility of applying a plant extract as an adjuvant which could augment an action of an acknowledged agent was also studied on an example of silymarin. Azim et al. [19] investigated effects of silymarin alone, *Moringa peregrina* leaf extract alone, and coadministration of those substances in rats subjected to acetaminophen. Generally, as regards plasma liver injury markers and oxidative parameters, the protective influence of all three treatments was found to be practically complete, but in some cases, the combination of both agents exerted the best effect. Interestingly, DNA damage was most markedly alleviated by plant extract alone while silymarin showed the slightest efficacy.

### 3.5. Immunosuppressive and Anti-Inflammatory Drugs vs. Plant Preparations

*Pistacia weinmannifolia* root extract was compared regarding anti-inflammatory effects with roflumilast—a drug used in the therapy of lung inflammatory disorders, in mice with pulmonary inflammation induced by cigarette smoke and lipopolysaccharide. The results of the experiment showed that the studied preparation possessed protective properties absolutely comparable with the applied medicine [149].

Sundaram et al. [55] studied the protective effect of guggulipid (an extract from *Commiphora whighitii* gum resin) against morphology changes, cartilage degradation, and prooxidative processes in rats with experimental arthritis. Along with the evaluation of the plant preparation properties, the authors performed the comparison with a standard nonsteroidal anti-inflammatory drug ibuprofen. The influence of the studied extract proved to be comparable or even much more effective, particularly in cases of hematological and oxidative parameters.

In a study performed on mice subjected to 1-chloro-2,4-dinitrobenzene with the aim of inducing atopic dermatitis-like skin lesion, the effect of *Rumex japonicus* Houtt. root extract was compared with the efficacy of a synthetic glucocorticoid dexamethasone. The alleviation of the disease severity caused by the topical application of the plant extract was not much worse (particularly in the case of the higher dose) than that observed in animals treated intraperitoneally with dexamethasone [77].

Kaveh et al. [117] in turn compared the influence of different doses of hydroethanolic extract of *Portulaca oleracea* with that exerted by dexamethasone in rats with experimental asthma. According the authors, the effect of the highest dose (4 mg/mL in drinking water) was comparable with the drug. However, the lowest dose (1 mg/mL in drinking water) in some cases showed no beneficial influence.

In some cases, the pharmaceutical agents showed a better effect. Sdayria et al. [41] reported that in mice with carrageenan-induced paw edema pretreatment with a nonsteroidal anti-inflammatory drug indomethacin or *Euphorbia retusa* methanol extract showed comparable effects concerning % edema inhibition, although indomethacin displayed a little better properties. However, in case of oxidant parameters in the liver and paw, the drug exerted more distinct beneficial influence.

In the experiment performed by Jeong et al. [48], a nonsteroidal anti-inflammatory drug celecoxib proved to be much better in reversing the changes of biochemical parameters observed in rats with monosodium iodoacetate-induced osteoarthritis than a water extract of leaves of *Morus alba* L. Histological examinations confirmed the drug advantage.

### 3.6. Standard Drugs vs. Plant Preparations in Stomach Ulcer Animal Model

Sattar et al. [137] performed a comparison of protective action of *Myristica fragrans* extract and sucralfate (a drug used for stomach ulcer treatment) in rats with ethanol-induced gastric ulcers. Although the plant preparation was not so effective with regard to amelioration of total acidity of stomach contents as well as macroscopic evaluation of gastric mucosa, ulcer index, and percentage of protection, its application did not cause serious increase in pH of stomach content as sulfacrate (4.25 vs. 5.0).

On the other hand, *Biebersteinia multifida* hydromethanolic extract showed a quite comparable or better protective effect than another drug—namely, omeprazole—in cases of gastric ulcers caused by 75% ethanol in rats. This effect included a decrease in ulcer area and number as well as enhancement of total antioxidant capacity in gastric mucosa [134].

Ateufack et al. [150] compared the effect of *Piptadeniastrum africanum* stem bark aqueous and methanol extracts (125, 250, or 500 mg/kg) in rat gastric ulcer induced by the HCl/ethanol mixture, indomethacin, and acetic acid with that showed by standard drugs (Maalox, Misoprostol, or Ranitidine). Plant extracts, particularly aqueous one when applied in the highest dose, displayed the protective action even better than the investigated drugs in case of animals exposed to HCl/ethanol mixture or indomethacin, but not in those treated with acetic acid.

Rtibi et al. [135] reported that in rats exposed to ethanol, pretreatment with *Ceratonia siliqua* L. aqueous extract (500, 1000, and 2000 mg/kg b.w.) showed a better or comparable effect with the standard drug famotidine when applied at the highest dose, while the lowest one was found to be either less effective or even ineffective at all.

The interesting results were reported by Chanda et al. [45]. The scientists studied the possibility of using *Paederia foetida* Linn. leaf methanol extract (100 and 200 mg/kg b.w.) as a gastroprotective agent in rats with gastric ulcers induced by indomethacin-pylorus ligation, alcohol, or water immersion stress. The effects were compared with those obtained for standard drugs ranitidine, sucralfate, and lansoprazole, respectively. As concerns ulcer protection, in the first and third models, there were no distinct differences between the plant material (particularly the higher dose) and the applied drug. In contrast, in the second model, the higher dose of extract showed much better effectiveness than the lower one, but nonetheless not so high as sucralfate.

### 3.7. The Comparison of Plant Preparations with Cytoprotective Adjuvants Used in Radio and Chemotherapy

The advantage of plant origin substances over drugs was also shown by Dong et al. [70]. Ethanol extract of JXT (a traditional herb obtained from the *Spatholobus suberectus* Dunn dry rattan) given orally to mice caused a significant improvement of the biochemical parameters previously disturbed by ^60^Co *γ*-radiation, i.e., morphology, bone marrow cell number, and liver lipid peroxidation level as well as activity of antioxidant enzymes. This effect was comparable or better than that exerted by amifostine, an agent applied in cases of radiation syndrome during radiotherapy.

### 3.8. Plant Preparations vs. Drugs and Supplements Applied in Neurological and Psychiatric Disorders

Pharmaceutical agents used for the treatment of psychiatric and neurodegenerative disorders may cause side effects worsening the condition of patients. Plant preparations, often used for centuries in traditional medicine, have been found to possess anxiolytic and antistress properties, and the comparison with acknowledged drugs and supplements showed their comparable efficacy [17, 58].

Plant materials, namely, extracts from two *Hypericum* species, were studied with regard to their effect on oxidative stress and inflammatory cytokines in an experimental anxiety animal model. The comparison with pure quercetin and a control drug alprazolam was also performed. In most cases, the disturbed brain parameters were positively influenced in a comparable or more distinct way by plant materials than by two other studied substances. It should be emphasized that in some cases the worst effect was observed in animals treated with alprazolam [17].

Almeida et al. [58] compared the protective effect of *Clitoria ternatea* extract with that shown by cotreatment with dietary supplements choline and docosahexanoic acid against brain oxidative stress caused by separation from mothers in rat pups. During 30-day experiment including the stressing factor and treatments as well as during the subsequent 330-day follow-up, both agents revealed comparable properties regarding the prevention of lipid peroxidation increase and thiol group content depletion.

Chonpathompikunlert et al. [31] compared the neuroprotective effect of *Apium graveolens* L. extract with that showed by a standard drug Tidomet Plus in an animal model of Parkinson disease. The efficacy of two doses (250 and 375 mg/kg b.w.) proved to be quite comparable (the lower one) or even better (the higher one) with the medicine as regards improvement of behavioral performance, oxidative parameters, and activities of monoamine oxidases A and B.

### 3.9. Antidiabetic Drugs vs. Plant Preparations

The possibility of plant preparations application in diabetic subjects was also studied. It was prompted by side effects occurring in patients treated by pharmacological agents. The outcomes seem to be promising although in view of the reported results the accurate research is needed to determine mechanism of action and the most beneficial dose [66, 127].

Khanra et al. [129] stated that *Abroma augusta* L. leaf methanol extract (100 or 200 mg/kg) was not so efficient at reversing the serum parameters disturbed in rats by type 2 diabetes course, particularly in the case of the lower dose, as a standard drug glibenclamide. However, in the case of alleviating DNA fragmentation, ATP level, chosen oxidant parameters, and expression of NF-*κ*B in the kidney and heart, the prevalence of the drug was not so distinct.

The comparison of glibenclamide and methanolic extract of *Caralluma europaea* was performed by Dra et al. [127] on diabetic mice. The higher dose of plant material (500 mg/kg b.w.) was better effective in the reduction of blood glucose than a drug, beginning from the 4^th^ hour after administration, while a lower one (250 mg/kg b.w.) showed a comparable effect. Additionally, according to the authors, the lower dose showed a more distinct beneficial influence in case of histopathological damage observed in diabetic animals.

The similar results were obtained by Du et al. [66] who compared the protective properties of a standard drug metformin hydrochloride and different doses (100, 250, and 500 mg/kg) of polysaccharide separated from *Lycium barbarum* in the rat model of diabetes induced by high-fat diet+streptozotocin. Metformin showed a better effect in case of fasting blood glucose and INF-*α*. As for insulin and ICAM-1, the highest dose of plant origin material proved to possess better ameliorating properties, while serum GPx was more improved by two higher doses.

Balbaa et al. [57] investigated the differences in effects of administration of *Nigella sativa* seeds oil, standard drugs metformin and glimepiride, and their combinations to diabetic rats. The oil had a better protective action when administrated alone than in combination with metformin or glimepiride against oxidative stress and neuroinflammatory cytokines' increase. When the results of administration of those three agents alone to diabetic rats were compared, the best properties of a plant material were also confirmed.

### 3.10. Plant Extract or a Single Substance of Antioxidant Properties?

As beneficial effects of plant preparations were attributed to their antioxidative properties, some researches performed the comparisons with simple substances of acknowledged antioxidant character. The results showed that plant materials could exert a better influence due to the presence of many component which might cooperate with one another.

Jahan et al. [72] studied the possibility of application of *Chenopodium album* Linn. seed extract as a protective agent against the damage of reproductive functions caused by mercury exposure. They compared the effect of the plant extract with that showed by a known antioxidant vitamin C which was reported to exert beneficial influence on male fertility. Except for plasma cholesterol and triglycerides as well as GST and TBARS in testicular tissue, the benefit influence of the studied plant material was comparable or even better. The similar observations were reported as regards the testicular morphometric parameters.

The comparison of the protective influence of *Nigella sativa* extract and vitamin E against cisplatin nephrotoxicity was performed by Hosseinian et al. [16]. The cisplatin-induced negative changes, i.e., renal damage and thiol group decrease, and lipid peroxidation enhancement in the serum and kidney, were alleviated in a rather comparable way, except for serum thiols, where the prevalence of plant extract was indisputable.

The effects of two *Hypericum* (*H. maculatum* and *H. perforatum*) species extracts on oxidative stress and inflammatory cytokines were studied in an experimental anxiety animal model and compared with pure quercetin. The beneficial influence of plant extracts was mostly comparable and in many cases better, particularly in the case of *Hypericum maculatum* [17]. Such results could point to synergistic action of various components of extracts, acting as confirmation of the conclusions made by He et al. [148].

### 3.11. The Action of a Plant Preparation Depending on Its Dose, Time and Form of Treatment

#### 3.11.1. The Relationships between the Dose and the Effects

In many studies, the protective effects of plant extracts and constituents showed a direct dependency on the used dose [15, 29, 31, 56, 80, 107, 117].

However, sometimes, the similar effects were observed for a considerable wide range of the applied doses. Liu et al. [91] studied an effect of various doses (0.0625, 0.125, 0.25, 0.5 g/kg b.w.) of ginseng oligopeptides against ethanol toxicity, and the observed differences were quite slight considering the size of the applied range.

Malik et al. [39] performed a study on animals with Huntington's disease like symptoms and observed practically the same influence of two different doses (100 and 200 mg/kg) of *Celastrus paniculatus* seed ethanol extract on memory functions, locomotor activity, and oxidative parameters in the brain parts.

The relationships between used doses and an exerted influence were also studied in the experiment concerning the protective properties of water extract of *Sonneratia apetala* fruit against liver damage caused by acetaminophen exposure in mice. The effects of pretreatment with different doses (100, 200 and 400 mg/kg) were very interesting as in the case of some parameters a strong dose dependency was observed (e.g., liver GSH and MDA), while some other ones were affected in the same way, regardless of the applied dose (liver GSH, T-AOC, and TNF-*α*) [51].

In another study, Wang et al. [119] investigated the protective properties of two doses (4.6 or 14 g/kg b.w.) of aqueous extract of Salvia Miltiorrhiza Bge. f. alba in the monocrotaline-induced animal model of pulmonary hypertension. Various parameters were studied: mean pulmonary artery pressure, right ventricular systolic pressure, pulmonary artery remodelling, plasma vasoactive factors NO, 6-Keto-PGF1*α*, ET-1, and TXB_2_, and lung TGF-*β*1, but the observed differences were not as considerable as one could expect taking into account the difference between the applied doses.

The similar observations were reported by El-Hadary and Ramadan [52] who studied two doses of *Moringa oleifera* leaf extract (150 and 300 mg/kg b.w.) against hepatotoxicity of diclofenac sodium. Both doses displayed protective properties, but again, the difference between them was not so great as one could predict considering their range.

Onyenibe et al. [122] studied the efficacy of two doses of *Monodora myristica* aqueous extract at preventing impairment of lipid profile and oxidative parameters in hypercholesterolemic rats and generally found no difference between 100 mg/kg b.w. and 200 mg/kg b.w.

Such results point to the necessity of complex research of any possible protective agent with using a wide range of doses. The issue of dependency between the used dose and its effects proved to be additionally complicated as not always a direct relationship was found. The effects of different doses were sometimes divergent as in the experiment performed by Khan et al. [88], who studied the influence of two doses 0.5 g/kg b.w. and 1.0 g/kg b.w. of ajwa dates (*Phoenix dactylifera* L.) water extract on the biochemical parameters in rats with diethylnitrosamine-induced liver cancer. The lower one in some cases showed even a harmful effect (potentializing the changes caused by the carcinogen), while the higher one showed considerable protective properties. Additionally, the lower dose effect was characterized by a significant diversity as apart from showing the above-mentioned harmful influence, it also exerted a beneficial action, ranging from slight to strong.

In some cases, the higher dose had the worse effect than a lower one. In the study performed by Omole et al. [26], concerning the possible use of pretreatment with kolaviron (a mixture of flavonoids obtained from *Garcinia kola* seeds, 200 or 400 mg/kg/d) against toxicity of cyclophosphamide, the higher dose proved to be less beneficial, not only exerting less protective properties but also causing a slight increase in lipid peroxidation in the heart tissue.

The similar observations were reported by Apaydin Yildirim et al. [28] who studied the influence of *Helichrysum plicatum* DC. subsp. *plicatum* extract (100 or 200 mg/(kg·d) against nephrotoxic as well as hepatotoxic effects of gentamicin in rats. The authors stated that the higher dose showed much less beneficial influence. Additionally, the higher dose exerted some harmful effects, particularly regarding histopahological changes, when administered alone.

In some studies, it would be really very difficult to decide which dose should be chosen for usage. In the experiment performed by Wattanathorn et al. [42], the beneficial influence of the combination of extracts of *Mangifera indica* L. and *Polygonum odoratum L*. against diabetes cataract and retinopathy was distinctly showed, but the effect of different doses (2, 10, or 50 mg/kg b.w.) was found to be highly diverse.

Phunchago et al. [92] studied the possible protective effect of *Tiliacora triandra* water extract against ethanol-induced hippocampus damage in rats. The authors compared three doses: 100, 200, and 400 mg/kg b.w., and found that the middle one showed the best influence in improving some studied parameters while in few cases the worst influence was found for the highest one.

#### 3.11.2. The Relationships between the Period of Treatment and the Effects

The period of the treatment with the studied substances also proved to be a factor of importance. Xu et al. [60] investigated the effect of Rhubarb extract on the oxidative parameters in rats with traumatic brain injury. According to the reported results, the degree of improvement in lipid peroxidation intensity as well as antioxidants levels showed a dependency on the time of experiment. The animals sacrificed after a longer period, starting from surgery and plant material treatment, showed a better amelioration of the studied parameters.

#### 3.11.3. The Relationships between the Way of Preparing Plant Materials and Their Effects

As plant extracts contain many active substances of different solubilities in various solvents, the way of the preparation of the used substances also proved to be an issue of importance.

Malik et al. [39] studied the fractions of *Celastrus paniculatus* seed ethanol extract, obtained by suspending it in water and sequential partitioning with using petroleum ether, ethyl acetate and n-butanol. These materials were investigated as to their ability to ameliorate the neurotoxic effect of 3-nitropropionic acid by reversing changes of oxidative parameters in striatum and cortex of experimental rats. In the case of improvement of enhanced MDA and nitrites as well as decreased CAT, SOD, and GSH, ethanol extract and aqueous fraction proved to be the most effective; the petroleum ether fraction showed much less efficacy while n-butanol and ethyl acetate ones practically none.

#### 3.11.4. Treatment of Pretreatment?

The way of treatment, i.e., pre or post, in some cases was shown to be a crucial factor influencing the observed effect to a considerable degree. Afsar et al. [23] studied the effects of ethyl acetate fraction of *Acacia hydaspica* methanol extract against cisplatin toxicity and observed that the protective influence observed in the case of posttreatment started concomitantly with cisplatin and continued for five days was decidedly less distinct than that of 15-day pretreatment combined with posttreatment.

On the other hand, some authors did not report any differences between the mentioned two ways. Khattab et al. [146] did not observe any differences between pre- and posttreatment with *Borago officinal* L. seed oil in rats exposed to *γ*-radiation.

The similar effects were observed by Nasri et al. [109] in rats subjected to a contrast medium iodixanol and 70% ethanol green tea extract. The plant agent alleviated iodixanol-induced histopathological kidney changes, but no significant differences between pre- and posttreatment were observed.

Hosseinian et al. [16] studied the possible protective effect against cisplatin nephrotoxicity of *Nigella sativa* extract (100 and 200 mg/kg) using the design, whereby two ways of administration were applied—pretreatment alone or with the addition of posttreatment. The outcomes were really interesting. In case of renal SH groups and tissue damage as well as serum SH groups and MDA plant material proved to possess beneficial effect, regardless of the way of administration.

## 4. The Effects of Plant Preparations Per Se

The fact that generally effects of plant preparations *per se* were observed occasionally makes the considerable limitation of the studies presented in the current review. Only a few scientists reported observations considering any influence of the studied materials. Sheweita et al., for instance, investigated the effects of application of essential oils of *Foeniculum vulgare* (fennel) Miller seeds, *Cuminum cyminum* L. (cumin) seeds, and *Syzygium aromaticum* L. (clove) flower and reported that the used oils themselves caused several beneficial effects like decrease in liver TBARS as well as enhancement of liver antioxidants [101].

El-Kashlan et al. [143] found the decrease in lipid peroxidation as well as reinforcement of antioxidant defence in rats receiving commercial date palm pollen.

El-Rahman et al. [110] reported alleviation of prooxidative processes as well as the increase in antioxidants in rats given *Saussurea lappa* root extract.

Furthermore, the beneficial influence of plant preparations included not only improvement in biochemical parameters. Sheng et al. [33] reported that, apart from positive effect on body and adipose tissue weight and insulin sensitivity, liver, and kidney functions, addition of mulberry leaves powder to diet also caused amelioration of microbiota community structure in gut of obese mice.

Balbaa et al. [57] investigated the differences in the effects of administration of *Nigella sativa* seeds oil, antidiabetic drugs metformin and glimepiride, and their combinations to rats. In some cases, the plant oil per se showed the least or no negative effect in nondiabetic rats when compared to medicines. The reduction of brain *β*-amyloid-42 as well as the increase in antioxidants' level was observed. On the other hand, brain lipid peroxidation was found to be enhanced.

However, some authors reported the negative effects of plant preparations on experimental animal organisms.

Apaydin Yildirim et al. [28] observed histopathological changes in organs of animals treated with *Helichrysum plicatum* DC. subsp. *plicatum* extract.

In one of the recently published articles, Nahdi et al. [151] observed that leaf *Hypericum humifusum* aqueous (200 or 400 mg/kg b.w.) and methanolic (10 or 20 mg/kg b.w.) extracts, given to rats, induced histopathological changes as well as impaired biochemical parameters including an increase in WBC, liver MDA, plasma ALT, AST, and LDH. Additionally, activities of hepatic antioxidant enzymes CAT and SOD were markedly decreased vs. control with no treatment. Interestingly, in case of the aqueous extract, the worse effect was exerted by the lower dose while the methanolic one was found to be more harmful when given in the higher dose.

## 5. Conclusions

The outcomes of the studies presented in the current review showed a huge potential inherent in plant preparations. They were revealed to reverse or alleviate toxicity of different factors, side effects of drugs, and symptoms of various diseases. In many cases, they were proved to be comparable or better than standard drugs which let us suggest that in future the plant origin substances could make a replacement for pharmaceutical agents. However, the presented above results of some experiments point to the fact that the proper precautions must be undertaken before applying any plant material. The detailed research regarding the *per se* effects, dose, and way of administration needs to be performed.

## Figures and Tables

**Table 1:** The protective properties of plant extracts against toxicity of substances applied in agriculture.

Reference	Plant preparation, dose, way and time of treatment, animals	The toxic substance, dose, way and time of exposure, and negative effects	Protective effects of plant preparation	Effects of plant *per se*
Chaâbane et al. [6]	*Nitraria retusa* fruit aqueous extract Pretreatment for 6 days and treatment during penconazole exposure 300 mg/kg b.w., p.o., daily Male Wistar rats about 250 g	A fungicide penconazole-induced kidney injury 67 mg/kg b.w. (1/30 LD50) i.p., every 2 days from 7^th^ until 15^th^ day Plasma: GGT, ALP ↓; CR, urea, UA, LDH ↑ Urine: volume ↑, CR, urea, UA ↓ Kidney: LDH, MDA, H_2_O_2_, PC, AOPP, NP-SH, GSH, MT, CAT, SOD, GPx ↑ Kidney: enlarged Bowman's space, necrosis of the epithelial cells lining the tubules, infiltration of leucocytes, glomeruli fragmentation ↑	Plasma: ALP (+), urea, LDH, GGT (++), CR, UA (+++) Urea: UA (+), volume, CR, urea (++) Kidney: LDH, MDA, H_2_O_2_, PC, AOPP, NP-SH, GSH, CAT, SOD, GPx (++), MT (+++) Kidney: necrosis of the epithelial cells lining the tubules (++), enlarged Bowman's space, infiltration of leucocytes, glomeruli fragmentation (+++)	Plasma: urea, LDH ↑ Urine: volume ↑, CR, urea ↓

Alzahrani et al. [15]	Standardized *Tribulus Terrestris* extract tablets 1000 mg (min. 45% saponins) Now Sports Co. (USA) 5 or 10 mg/kg daily, p.o., for 17 days Male Swiss albino mice, 20-28 g	A pesticide rotenone-induced parkinsonism, nine doses of 1 mg/kg given each 48 ± 2 h, s.c. Activity index (18^th^ day) ↓ Substantia nigra: % of pycnotic neurons ↑, % of viable neurons ↓ Striatum: relative expression of iNOS, COX-2 and MTH 1, MDA, 8-OH-dG ↑, dopamine, GSH, SOD, CAT ↓	Activity index (18^th^ day) (+ 5, +++ 10) Substantia nigra: % of viable neurons (+ 50, +++ 10), % of pycnotic neurons (++ 5, +++ 10) Striatum: GSH, SOD, MDA (0 5, ++ 10), CAT (0 5, +++ 10), dopamine (+ 5, ++ 10), relative expression of COX-2 and iNOS (++ both doses), 8-OH-dG and relative expression of MTH 1 (++ 5, +++ 10)	None

Selmi et al. [71]	*Lavandula stoechas* essential oils 50 mg/kg b.w., p.o., for 30 days Male mice, 8-week old, 25-30 g	Malathion (a pesticide) 200 mg/kg b.w./day for 30 days, p.o. Serum: T ↓ Testis: relative weight, MDA, H_2_O_2_ ↑, -SH groups, GPx, CAT, SOD total, Cu/Zn-SOD, Mn-SOD ↓ Epididymis: relative weight, MDA, H_2_O_2_ ↑, -SH groups, GPx, CAT, SOD total, Cu/Zn-SOD, Mn-SOD ↓	Serum: T (+++) Testis: Mn-SOD (0), -SH groups, SOD total (++), relative weight, MDA, H_2_O_2_, GPx, Cu/Zn-SOD, CAT (+++) Epididymis: CAT and H_2_O_2_ (++), relative weight, MDA, -SH groups, GPx, SOD total, Cu/Zn-SOD, Mn-SOD (+++)	Testis: CAT ↑

El Arem et al. [7]	*Phoenix dactylifera* L. date palm fruit aqueous extract 4 mL/kg daily, p.o., for 2 months Male Wistar rats 180–200 g	Dichloroacetic acid (a fungicide) 0.5 g/L or 2 g/L as drinking water, for 2 months 0.5 g/L: Weight: testes, epididymis ↓ Plasma: T, FSH, LH ↓ Testes: CAT, SOD, LPO ↑, GSH ↓ 2 g/L: Weight: testes, epididymis ↓ Plasma: T, FSH, LH ↓ Testes: CAT, SOD, LPO ↑, GSH, GPx ↓	0.5 g/L of dichloroacetic acid: Weight: testes, epididymis (+++) Plasma: T, FSH, LH (+++) Testes: CAT, SOD, LPO, GSH (+++) 2 g/L of dichloroacetic acid: Weight: testes (++), epididymis (+++) Plasma: T, FSH, LH (++) Testes: CAT, SOD, GSH, LPO (++), GPx (+++)	None

Mossa et al. [9]	*Vitis vinifera* grape pomace (El Kroom Company, Alexandria, Egypt) 80% ethanolic extract 100 or 200 mg/kg b.w., p.o., 28 consecutive days Weanling female rats about 50 g	Cypermethrin (an insecticide) 25 mg/kg b.w. (1/10 of LD50), p.o., for 28 consecutive days Relative weight: liver ↓, kidney ↑ Serum: TP, ALB ↓, AST, ALT, ALP, GGT, urea nitrogen, CR ↑ Liver: Kupffer cell activation, portal infiltration with inflammatory cells, hyperplasia of bile duct, congestion of central vein and hepatic sinusoids ↑ Kidney: vacuolization of endothelial lining glomerular tuft, vacuolization of epithelial lining renal tubules, necrosis of epithelial ↑	Relative weight: liver, kidney (+++ both doses); Serum: ALT, CR (++ both doses), AST, ALP, GGT, TP (++ 100, +++ 200), ALB, urea nitrogen (+++ both doses) Liver: Kupffer cell activation (+ 100, ++ 200), portal infiltration with inflammatory cells, hyperplasia of bile duct, congestion of central vein and hepatic sinusoids (+++ both doses) Kidney: vacuolization of endothelial lining glomerular tuft (++ 100, +++ 200), vacuolization of epithelial lining renal tubules, necrosis of epithelial (+++ both doses)	Both doses: Liver: Kupffer cell activation ↑

Mirzaei et al. [2]	*Quercus brantii* 70% ethanol extract of internal layer of the fruit 500 mg/kg, p.o., for 9 days Male Wistar albino rats, 150-200 g	Carbendazim: methyl-2-benzimidazole carbamate (a fungicide agent), 50 mg/kg p.o., for 9 days Serum: ALT, AST, ALP, urea nitrogen, CR ↑ Liver: MDA ↑, GSH ↓ Kidney: MDA ↑, GSH ↓	Serum: ALT, AST, ALP, urea nitrogen, CR (++) Liver: MDA (+), GSH (++) Kidney: MDA, GSH (++)	Not studied

Khalaf et al. [8]	*Punica granatum* L. pomegranate peel methanol extract 250 mg/kg daily, i.g., for 4 weeks Male Wistar rats, 140-160 g	Gibberellic acid-3 (a plant growth regulator) 20 mg/kg i.g., daily for 4 weeks Testes: area percent of androgen receptor immunoreaction, SOD, CAT ↓	Testes: area percent of androgen receptor immunoreaction, SOD, CAT (+++)	None

↓: a decrease vs. control; ↑: an increase vs. control; (+): a slight beneficial effect; (++): a distinct beneficial effect; (+++): a complete beneficial effect; (0): no beneficial effect.

**Table 2:** The protective effects of plant origin substances against toxicity of heavy metals.

Reference	Plant preparation, dose, way and time of treatment, animals	The toxic substance, dose, way and time of exposure, and negative effects	Protective effects of plant preparation	Effects of plant *per se*
Abdel Moneim [10]	*Indigofera oblongifolia* leaves water-methanol (1 : 2) extract 100 mg/kg b.w. p.o. daily, 1 h before Pb for 5 days Male Wistar rats, 8-week-old, 150–180 g	Pb lead acetate acute toxicity, 20 mg/kg b.w. daily for 5 days, i.p. Serum: AST, ALT, ALP, total bilirubin, TC, TG, LDL-c ↑, HDL-c ↓ Liver: relative weight, Pb accumulation, LPO, NO and H_2_O_2_, ↑, GSH, SOD, CAT, GPx, GR ↓ Liver: expression of mRNA of SOD2, CAT, GPx and Bcl-2↓, Bax and HO-1↑	Serum: ALP, total bilirubin, TG, HDL-c (++), AST, ALT, TC, LDL-c (+++) Liver: relative weight, Pb accumulation, NO, H_2_O_2_, SOD, CAT, GR (++), LPO, GSH, GPx (+++) Liver: HO-1(-), expression of mRNA of SOD2, CAT, GPx, Bax, Bcl-2 (++)	Liver: GSH, GPx ↑

Long et al. [62]	Proanthocyanidins extracted from grape seeds (Zelang Medical Technology Company, Nanjing, China) 100 mg/kg b.w., p.o., six days every week for 6 weeks Male Kunming mice, 3-week-old	Pb lead acetate 0.2% in drinking water for 6 weeks Blood: Pb ↑ Serum: ALT, AST, ALP ↑ Liver: GSH, GPx and SOD ↓, relative mRNA expression of Nrf2, *γ*-GCS, HO-1 slightly ↑, Pb and MDA as well as relative mRNA expression of Bax, GRP78 and CHOP ↑, relative mRNA expression of Bcl-2 ↓	Blood: Pb (0) Serum: ALT, AST, ALP (++) Liver: Pb (+), GPx, SOD, relative mRNA expression of Bax, Bcl-2 GRP78, CHOP (++), MDA, GSH (+++), relative mRNA expression of Nrf2, *γ*-GCS, HO-1 ↑	Liver: relative mRNA expression of Nrf2, *γ*-GCS, HO-1 ↑

El-Boshy et al. [11]	*Thymus vulgaris* leaf ethanol extract 500 mg/kg/day, p.o., for 6 weeks Sprague-Dawley male rats, about 150 g	Pb lead acetate 500 mg/L in drinking water, for 6 weeks Serum: TNF-*α*, IL-1*β*, IL-6 ↑, INF-*γ*, IL-10 ↓ Liver: Pb, MDA ↑, GSH, GPx, CAT ↓ Kidney: Pb, MDA↑, GSH, GPx, CAT↓	Serum: IL-1*β*, IL-6 (++), INF-*γ* and IL-10 (+++) Liver: Pb (++), MDA, GSH, GPx, CAT (+++) Kidney: Pb (++), MDA, GSH, GPx, CAT (+++)	Serum: IL-10 ↑ Liver: GSH, GPx ↑ Kidney: GSH, GPx ↑

Dua et al. [3]	*Ipomoea aquatica* and *Enhydra fluctuans* aerial parts aqueous extracts Pretreatment 100 mg/kg b.w., p.o., for 5 days followed by cadmium chloride Swiss male albino mice, 1-2 months, 20-30 g	Cd cadmium chloride, 4 mg/kg b.w., for 6 days, once daily, p.o. Blood: RBC, HGB ↓ Serum: ALT, AST, urea, TC, TG ↑, HDL-c ↓ Liver: cadmium, ROS, LPO, NADH oxidase, protein carbonylation ↑, total coenzymes Q9 and Q10, CAT, SOD, GST, GPx, GR, G6PD, GSH ↓ Kidney: cadmium, ROS, LPO, NADH oxidase, protein carbonylation ↑, total coenzymes Q9 and Q10, CAT, SOD, GST, GPx, GR, G6PD, GSH ↓ Heart: cadmium, ROS, LPO, NADH oxidase, protein carbonylation ↑, total coenzymes Q9 and Q10, CAT, SOD, GST, GPx, GR, G6PD, GSH ↓ Brain: cadmium, ROS, LPO, NADH oxidase, protein carbonylation ↑, total coenzymes Q9 and Q10, CAT, SOD, GST, GPx, GR, G6PD, GSH ↓ Testes: cadmium, ROS, LPO, NADH oxidase, protein carbonylation ↑, total coenzymes Q9 and Q10, CAT, SOD, GST, GPx, GR, G6PD, GSH ↓	Both extracts: Blood: RBC, HGB (++) Serum: ALT AST, urea, TC, TG, HDL-c (++) Both extracts: Cadmium burden, ROS, G6PD, NADPH oxidase: liver, kidney, heart, brain, testes (++) LPO: liver, kidney, heart, brain, testes (+++); Protein carbonylation: liver, kidney, heart, testes (++), brain (+++) Total coenzyme Q9: liver, brain, heart, testes (++), kidney (+++) Total coenzyme Q10: kidney, brain, testes (++), liver, heart (+++) CAT: liver, brain, heart (++), kidney, testes (+++) SOD, GST: liver, kidney, heart, brain, testes (+++) GPx: liver, heart, testes (++), brain, kidney (+++) GR: heart, testes, brain (++), liver, kidney (+++) GSH: heart, kidney, brain (++), liver, testes (+++)	Not studied

Olaniyan et al. [1]	*Plukenetia conophora* seeds methanol extract 100 or 200 mg/kg b.w., p.o., for 54 days after CdCl_2_ treatment Male Wistar rats, 150-190 g	Cd cadmium chloride, a single dose 2 mg/kg b.w., i.p. Serum: T, LH, FSH ↓ Epididymal semen: count of cells, motility, viability ↓ Testis: MDA and NO ↑, SOD, CAT, GPx, GST, Na^+^/K^+^ATPase, Ca^2+^ATPase, Mg^2+^ATPase ↓ Epididymis: MDA and NO ↑, SOD, CAT, GPx, GST ↓	Serum: LH (0 100, + 200), FSH (+ both doses), testosterone (++ 100, +++ 200) Epididymal semen: count of cells, motility, viability (++ both doses) Testis: Mg^2+^ATPase (++ both doses), Na^+^/K^+^ATPase (++ 100, +++ 200), MDA, NO, SOD, CAT, GST (+++ both doses), Ca^2+^ATPase (+++ 100, ↑ 200), GPx (↑ both doses) Epididymis: GPx (+ both doses), SOD, GST, Na^+^/K^+^ATPase and Ca^2+^ATPase (++ both doses), CAT (++ 100, +++ 200), MDA, Mg^2+^ATPase (+++ both doses), NO (+++ 100, ↓ 200)	Not studied

Mężyńska et al. [5]	*Aronia melanocarpa* L. berries extract (Adamed Consumer Healthcare, Tuszyn, Poland) 0.1% aqueous solution in form of the only drinking fluid for 3, 10, 17 or 24 months Female Wistar rats, 3-4-week-old	Cd cadmium chloride in diet 1 mg/kg (Cd_1_) or 5 mg/kg (Cd_5_) for 3, 10, 17 or 24 months Liver: SOD (Cd_1_ 3, 10, 17 m) and (Cd_5_ 17 m) ↓; CAT (Cd_5_ 17 m) ↓; GPx (Cd_1_ 3,10,17,24 m), (Cd_5_ 3, 10, 17, 24 m) ↓; GR (Cd_1_ 10, 24 m), (Cd_5_ 10, 17 m) ↓; GST (Cd_1_ 3, 10, 17 m), (Cd_5_ 3, 10, 17 m) ↓; GSH/GSSG (Cd_1_ 17 m), (Cd_5_ 10, 17 m) ↓; H_2_O_2_ (Cd_1_ 10, 17, 24 m), (Cd_5_ 10, 17, 24 m) ↑; OSI (Cd_1_ 10, 17 m), (Cd_5_ 3, 10, 17, 24 m) ↑; MDA (Cd_1_ 10, 17, 24 m), (Cd_5_ 10, 17, 24 m) ↑	Liver: SOD: Cd_1_ (+ 3 m, 0 10 m, +++ 17 m), Cd_5_ (+++ 17 m) CAT: Cd_5_ (++ 17 m) GPx: Cd_1_ (+++ 3, 10 m, ++ 17, 24 m), Cd_5_ (0 3 m, ++ 10 and 17 m, +++ 24 m) ↓ GR: Cd_1_ (+++ 10 m, ↑ 24 m), Cd_5_ (++ 1 m, - 17 m) GST: Cd_1_ (+++ 3, 10, 17 m), Cd_5_ (++ 3 m, 0 10 m, +++ 17 m) GSH/GSSG: Cd_1_ (+++ 1 m), Cd_5_ (+++ 10 m, ↑ 17 m) H_2_O_2_; Cd_1_ (+++ 10, 17, 24 m), Cd_5_ (+++ 10, 17, 24 m) OSI: Cd_1_ (+++ 10, 17 m), Cd_5_ (+++ 3, 10, 17, 24 m) MDA: Cd_1_ (+++ 10, 17, 24 m), Cd_5_ (+++ 10, 17, 24 m)	Liver: CAT10m ↓, GR 3 m ↓, GSH/GSSG 24 m ↑

Ke et al. [12]	*Pyracantha fortuneana* fruits 60% ethanol extract Pretreatment, 1 h before cadmium, 250 mg/kg, p.o., for 5 days Male Wistar rats, 7-8-week-old, 150-170 g	Cd cadmium chloride, 6.5 mg/kg daily for 5 days, i.p., Body weight gain ↓ Kidney weight ↑ Plasma: UA, urea and CR ↑ Kidney: Cd accumulation, MDA, NO, expression of Keap1, Bax and TNF-*α* protein ↑, CAT, GPx, GSH, SOD, GR, expression of Bcl-2, HO-1, *γ*-GCS, NQO1 and Nrf2 protein ↓	Body weight gain (++) Kidney weight: (++) Plasma: urea, UA and CR (++) Kidney: Cd accumulation, MDA, NO, CAT, GPx, GSH, SOD, GR (++), expression of Bcl-2, Bax and TNF-*α* protein (++), expression of Keap1, HO-1, *γ*-GCS, NQO1 and Nrf2 protein ↑	Kidney: expression of Keap1, HO-1, *γ*-GCS, NQO1, Nrf2 ↑

Kim et al. [73]	*Dendropanax morbifera* leaf 80% ethanol extract 100 mg/kg, p.o., daily for 4 weeks Male Sprague-Dawley rats, 7-week-old	Hg dimethylmercury daily 5 *μ*g/kg, i.p., for 4 weeks Hippocampus: Hg concentration, ROS production, GST, MDA ↑, SOD1, total sulfhydryl content, CAT, GPx, GR ↓	Hippocampus: CAT, total sulfhydryl content (+), Hg concentration, ROS production, MDA, SOD1, GST, GPx, GR (++)	None

Jahan et al. [72]	*Chenopodium album* Linn. seed ethanol extract 200 mg/kg b.w., p.o., for 30 days Sprague-Dawley male rats, about 240 g	Hg mercury(II) chloride, 0.15 mg/kg b.w. for 30 days, i.p. Body weight ↓ Plasma: BUN, CR, cholesterol, TG, LDL ↑, HDL, T ↓ Testicular tissue: TP, CAT, SOD, GST ↓, ROS and TBARS ↑ Daily sperm production: ↓	Body weight (+++) Plasma: cholesterol, TG (-), LDL, CR and HDL (++), BUN and T (+++) Testicular tissue: TP, CAT, SOD, GST, ROS and TBARS (++) Daily sperm production: (+++)	None

Gao et al. [4]	*Rheum palmatum* L. dried root and Rhizoma (Lixian Pharmaceuticals Company, Longnan, China) 60% ethanol extract 1200 mg/kg, i.g., daily for 7 days Sprague-Dawley male rats, 140–160 g	Hg mercury(II) chloride, a single dose of 2 mg/kg, s.c., on the 5^th^ day Body weight: ↓ Serum: TP, ALB ↓ BUN, CR ↑ Kidney: GSH, GPx and CAT ↓ Renal tubule epithelial cells: swelling, necrosis, granular degeneration, interstitial vascular congestion ↑	Body weight: (++) Serum: ALB, CR, BUN (++); TP (+++) Kidney: GPx, CAT (0), GSH (+) Renal tubule epithelial cells: swelling (+), necrosis granular degeneration, interstitial vascular congestion (++)	Not studied

Ghate et al. [50]	*Drosera burmannii* Vahl. 70% methanol extract 50, 100, or 200 mg/kg b.w., p.o., for 21 days from the next day after first injection of iron dextran Male Swiss mice, 20 g	Iron-dextran 100 mg/kg b.w., i.p., five doses (one dose every two days) Serum: ALT, AST, ALP, bilirubin, ferritin ↑ Liver: SOD, CAT, GST, GSH ↓, LPO ↑	Serum: bilirubin (+ 50, ++ 100 +++ 200), ALT and AST (++ all doses), ALP and ferritin (++ 50 and 100, +++ 200) Liver: SOD (0 50, ++ 100, +++ 200); CAT, GST, and LPO (+++ all doses); GSH (0 50, ++ 100 and 200)	Not studied

↓: a decrease vs. control; ↑: an increase vs. control; (+): a slight beneficial effect; (++): a distinct beneficial effect; (+++): a complete beneficial effect; (0): no beneficial effect; (-): intensification of the harmful effect.

**Table 3:** The protective effects of plant preparations against toxicity of different chemicals.

Reference	Plant preparation, dose, way and time of treatment, and animals	The toxic substance, dose, way and time of exposure, and negative effects	Protective effects of plant preparation	Effects of plant *per se*
AlSaid et al. [13]	*Piper cubeba* fruits 70% ethanol extract Pretreatment 250 or 500 mg/kg for 7 days, p.o. Male Wistar albino rats 180–200 g	CCl_4_-induced hepatotoxicity, a single dose of 0.4 mL/kg, i.p. Serum: ALT, AST, GGT, ALP, bilirubin, LDH ↑ Liver: MDA ↑, NP-SH, CAT, TP ↓ Liver: mRNA expression of TNF-*α*, IL-6, IL-10, HO-1, iNOS ↑	Serum: ALT, AST, LDH (0 250, ++ 500), GGT (+ 250, ++ 500), ALP, bilirubin (++ both doses) Liver: TP (0 250, ++ 500), MDA, CAT (+ 250, ++ 500), NP-SH (++ 250, +++ 500) Liver: mRNA expression of IL-10 (↑ both doses), TNF-*α*, IL-6, HO-1, iNOS (++)	Not studied

Bellassoued et al. [14]	*Mentha piperita* L. leaf essential oil Pretreatment 5, 15, and 40 mg/kg b.w., daily for 7 days, p.o. Male Wistar rats 200–220 g	CCl_4_: 1 mL/kg b.w. on the 7^th^ day, i.p. Serum: ALT, AST, ALP, LDH, GGT, TC, TG, LDL, urea, CR ↑, HDL ↓ Liver: LPO, inflammatory cells and cellular necrosis ↑, SOD, CAT, GPx ↓ Kidney: LPO, glomerular and epithelial cells of the proximal tubules necrosis ↑, SOD, CAT, GPx ↓	Serum: ALT, AST, ALP, LDH, GGT, TC, TG, HDL, LDL, urea, CR (+ 5, ++ 15, 40) Liver: inflammatory cells and cellular necrosis, LPO, SOD, CAT, GPx (+ 5, ++ 15, and 40) Kidney: LPO, SOD, CAT, GPx, glomerular and epithelial cells of the proximal tubules necrosis (+ 5, ++ 15, and 40)	40 mg/kg b.w. only studied: none

Simeonova et al. [54]	*Astragalus monspessulanus* L. n-butanolic extract—obtained by successive extraction of 80% methanol extract with CH_2_Cl_2_, ethyl acetate, and n-butanol Pretreatment 100 mg/kg, for 7 days, p.o. Male Wistar rats 200–220 g	CCl_4_-induced hepatotoxicity a single dose 1.25 mL/kg of 10% CCl_4_ in olive oil Serum: ALT, AST, ALP ↑ Liver: MDA ↑, GSH, CAT, SOD, GPx, GR, GST ↓	Serum: ALT, AST, ALP (+++) Liver: GR, GSH, CAT, GPx, GST (++), MDA, SOD (+++)	None

Shah et al. [80]	*Commelina nudiflora* L. methanol extract Pretreatment for 12 days and 2-day-treatment 150, 300, 450 mg/kg b.w., p.o. Male Sprague Dawley rats, 150–250 g	CCl_4_ 1.0 mL/kg b.w., two doses on 13^th^ and 14^th^ days Serum: ALT, AST ↑ Liver: GSH, GST, GPx, GR, CAT, G6PD, QR ↓, LPO, HNE-modified protein adducts, 8-OHdG, TNF-*α*, IL-6, PGE_2_ ↑	Serum: ALT, AST (++ all doses) Liver: GSH, GST, GR, CAT, G6PD, QR, LPO, HNE-modified protein adducts, 8-OHdG, TNF-*α*, IL-6, PGE_2_ (++ all doses), GPx (++ 150, +++ 300, 450)	450 mg/kg b.w. only studied: none

Yoshioka et al. [78]	*Sasa veitchii* leaf extract Pretreatment 0.2 mL (1 mL made from 2.82 g of leaves) for 7 days once per day, p.o. Male ddY mice, 7 weeks	CCl_4_ 3 g/kg a single injection, i.p. Plasma: ALT, AST, BUN, CR ↑ Liver: MDA, Ca ↑ Kidney: MDA ↑	Plasma: ALT, AST, BUN (++), CR (+++) Liver: MDA, Ca (++) Kidney: MDA (+)	None

Lin et al. [82]	*Chenopodium formosanum* Koidz (red quinoa): whole seed powder, 50% ethanolic bran extract, aqueous bran extract 5.13 g/kg of red quinoa whole seed powder (rutin 8.46 mg/kg/day), p.o. 1.54 g/kg of red quinoa bran 50% ethanol extract (rutin 16.4 mg/kg/day), p.o. 1.54 g/kg of red quinoa bran water extract (rutin 3.92 mg/kg/day), p.o. Male BALB/c mice	CCl_4_ 0.5 mL/kg b.w., two times weekly (Thursday and Sunday) for 6 weeks, i.p. Serum: AST, ALT, TP, ALB, globulins, bilirubin, CR, BUN ↑, ALP ↓ Liver: TBARS, ROS, TNF-*α*, TGF-*β*1, IL-6 ↑, SOD, CAT ↓	Whole seed powder: Serum: ALP (0), CR, BUN, ALB, globulins (+), ALT, TP, bilirubin (++), AST (+++) Liver: TBARS, ROS, TNF-*α*, TGF-*β*1, IL-6, SOD, CAT (+++) Ethanol bran extract: Serum: ALP (↓), ALB, globulins, BUN (+), CR, ALT, AST, TP, bilirubin (++) Liver: TGF-*β*1 (+), ROS (++), TBARS, TNF-*α*, IL-6, CAT (+++), SOD (↑) Water bran extract: Serum: BUN, ALP (0), CR, bilirubin, ALB, globulins (+), ALT, AST, TP (++) Liver: CAT, TGF-*β*1, ROS (+), IL-6 (++), TBARS, TNF-*α* (+++), SOD (↑)	Not studied

Parvez et al. [83]	*Solanum surattense* leaves 70% ethanol extract 100 and 200 mg/kg b.w., p.o., for 3 weeks Male Wistar rats, 8-9-week-old, 200-220 g	CCl_4_ in liquid paraffin (1: 1) 1.25 mL/kg b.w., i.p. Serum: AST, ALT, ALP, GGT, bilirubin, TC, TG, LDL, VLDL ↑; TP and HDL ↓ Liver: MDA ↑, NP-SH ↓	Serum: AST (0 100, + 200), TP, HDL (0 100, ++ 200), GGT, LDL, VLDL (+ 100, ++ 200) ALT, ALP, bilirubin, TC, TG (++ both doses) Liver: MDA (0 110, ++ 200), NP-SH (++ both doses)	Not studied

Bahcecioglu et al. [76]	*Pistacia terebinthus* coffee (coffee branded “Harput Çedene Coffee”) Freshly prepared coffee in drinking water (the content increased by 25% during each preparation, reaching up to 100% at the end of 7 days), for 8 weeks Male Sprague-Dawley rats about 250 g	Thioacetamide-induced chronic liver injury, 100 mg/kg b.w., i.p., three times weekly for 8 weeks Plasma: MDA↑ Liver: TNF-*α*, NF-*κ*B, TGF-*β* ↑ Liver: inflammation, necrosis, fibrosis ↑	Plasma: MDA (+) Liver: TNF-*α*, NF-*κ*B, TGF-*β* (++) Liver: inflammation, necrosis, fibrosis (++)	Liver: TGF-*β* ↓

Thomaz al. [84]	*Eugenia dysenterica* leaf hydroalcoholic extract 10, 100, 300 mg/kg/day, p.o., for 45 days starting from the 45^th^ day of AlCl_3_ administration Male Swiss mice, 25-30 g	AlCl_3_-induced neurotoxicity 100 mg/kg/day p.o., for 90 days Brain cortex: MDA ↑, SOD ↓ Hippocampus: MDA ↑, CAT, SOD ↓ Hippocampus CA1 area: % of viable neurons ↓, % of necrotic neurons ↑	Brain cortex: MDA and SOD (+++) all doses Hippocampus: CAT (0 10, +++ 100 and 300), MDA and SOD (+++ all doses) Hippocampus CA1 area: % of viable neurons (+++ 10, ++ 100, and 300), % of necrotic neurons (0 10, ++ 100, and 300)	Not studied

Feng et al. [74]	Defatted walnut meal (Shaanxi Sea Ecological Agriculture Co., Ltd., Shangluo, China) protein hydrolysates 1 g/kg per day, for 90 days, i.g., Male wild-type Kunming mice, 8 weeks	Neurotoxicity induced by D-galactose 200 mg/kg/day, s.c. (6 hours after plant material), and AlCl_3_ (100 mg/kg in the drinking water) for 90 days Brain: SOD, GPx, ChAT ↓, MDA, AchE, TNF-*α*, IL-1*β* ↑	Brain: SOD, GPx, ChAT (++), MDA, AchE, TNF-*α*, IL-1*β* (+++)	None

Kukongviriyapan et al. [81]	*Antidesma thwaitesianum* 95% ethanolic extract of the fruits pomace 100 or 300 mg/kg once daily, p.o. Male Sprague-Dawley rats, 200-220 g	N^*ω*-^nitro-L-arginine methyl ester-induced nitric oxide deficiency, 50 mg/kg/day in drinking water, for 3 weeks Blood pressure: SBP, DBP ↑ Plasma: MDA, PC ↑, NO_3_^−^/NO_2_^−^ level ↓ Carotid artery: superoxide formation ↑ Aortic tissue: eNOS protein expression ↓	Blood pressure: SBP, DBP (++ both doses) Plasma: PC (0 100, +++ 200), MDA, NO_3_^−^/NO_2_^−^ level (++ 100, +++ 200) Carotid artery: superoxide formation (++ 100, +++ 200) Aortic tissue: eNOS (+ 100, +++ 200)	None

Yang et al. [77]	*Rumex japonicus* Houtt. roots 95% ethanolic extract 4 mg/mL and 8 mg/mL, topically to the skin and ears, daily for 3 weeks Female Balb/c mice, 5-week-old	DNCB- (1-chloro-2,4-dinitrobenzene-) induced atopic dermatitis 200 *μ*L of 0.5% DNCB (in acetone-olive oil mixture) to the dorsal skin and ear, on days 1–3 Ear thickness ↑ Lymph nodes and spleen weights ↑ Skin: mast cell number ↑	Ear thickness: (++ both doses, a better effect for a higher dose) Weight of lymph nodes: (+ 4, ++ 8) Weight of spleen: (++ both doses) Skin: mast cell number (++ both doses, a better effect for a higher dose)	Not studied

Zheng et al. [85]	*Dracocephalum heterophyllum* 95% ethanol extract Pretreatment 2 h before Concanavalin A 20 mg/kg b.w., i.p. Female BALB/c mice, 6-8-week-old	Concanavalin A-induced hepatitis sublethal dose 15 mg/kg b.w., i.v. Serum 8, 16, and 24 hours after Concanavalin A: ALT, AST, IFN-*γ*, TNF-*α* ↑ Liver: MDSCs ↓, Kupffer cells ↑	Serum 8, 16, and 24 hours after Concanavalin A: ALT, AST, IFN-*γ*, TNF-*α* (++) Liver: Kupffer cells (+++), MDSCs ↑	Liver: Kupffer cells ↑

Badr and Naeem [75]	*Physalis peruviana* cape goldenberry fruit powder 20% (*w*/*w*) fruit powder addition to diet, for 35 days Male albino rats, 1 month, 140-150 g	Aflatoxins B_1_ and G_1_ 850 ng/kg b.w./day for 35 days Weight gain, food intake ↓ Blood: HGB, RBC, PLT ↓, HCT, WBC ↑ Serum: Fe, T-AOC, cholesterol ↓, TG, LDL-c, HDL-c, ALT, AST, ALP, MDA ↑ Liver: SOD, CAT ↓, MDA ↑	AFB_1_ Weight gain (++), food intake (+++) Blood: PLT, HCT, WBC, HGB (++), RBC (+++) Serum: MDA (+), LDL-c, HDL-c, Fe, cholesterol, T-AOC, TG, ALT, AST, ALP (++) Liver: SOD, MDA (++), CAT (+++) AFG_1_ Weight gain (++), food intake (+++) Blood: PLT, HCT, WBC, HGB (++), RBC (+++) Serum: LDL-c, HDL-c, Fe, cholesterol, TG, ALT, AST, ALP, MDA (++), T-AOC (+++) Liver: SOD, MDA (++), CAT (+++)	None

Xu et al. [79]	Polysaccharides from *Morinda officinalis* processed root (processed root by Beijing Sanhe Pharmaceutical Co., Ltd.) 500 mg/kg i.g., once a day for 18 days Female Balb/c mice	Concanavalin A-induced liver damage 10 mg/kg i.v., once a week Weight index: liver and kidney ↑, thymus ↓ Serum: ALT and AST ↑ Liver: CAT↓, MDA ↑	Weight index: kidney (0), liver (+), thymus (+++) Serum: ALT and AST (+++) Liver: CAT, MDA (+)	Not studied

Ahmed et al. [86]	*Pulicaria petiolaris* aerial part methanol extract Pretreatment 50 or 100 mg/kg, p.o., for 5 days Male Swiss albino mice, 25-27 g	LPS-induced hepato- and cardiotoxicity, a single dose 10 mg/kg i.p. Serum: ALT, AST, ALP, LDH, CK-MB, cTnI ↑ Liver: GSH, SOD ↓, MDA, NF-*κ*B, TNF-*α*, IL-6, average severity of histopathological lesions ↑ Heart: GSH, SOD ↓, MDA, NF-*κ*B, TNF-*α*, IL-6, average severity of histopathological lesions ↑	Serum: ALT, AST, ALP, CK-MB, cTnI (++ both doses), LDH (++ 50, +++ 100) Liver: average severity of histopathological lesions (+ 50, ++ 100), NF-*κ*B, (++ both doses), MDA, IL-6, TNF-*α*, GSH (++ 50, +++ 100), SOD (+++ both doses) Heart: IL-6, NF-*κ*B, TNF-*α*, GSH, SOD (++ both doses), average severity of histopathological lesions and MDA (++ 50, +++ 100)	100 mg/kg only studied: Serum: LDH ↓

Raish et al. [87]	*Lepidium sativum* seed ethanol extract Pretreatment 150 and 300 mg/kg p.o., for 14 days Male Wistar rats, 2-month-old, 180-205 g	D-Galactosamine+LPS-induced hepatotoxicity 400 mg/kg and 30 *μ*g/kg, respectively, i.p., on the 15^th^ day Serum: ALT, ALP, AST, bilirubin, GGT ↑ Liver: CAT, GSH, SOD ↓, MDA, MPO, NF-*κ*B9 (p65) DNA binding activity, mRNA expression of TNF-*α*, IL-6, IL-10, HO-1, iNOS ↑	Serum: ALT, ALP, AST, GGT (++ both doses), bilirubin (++ 150, +++ 300) Liver: CAT (+ 150, ++ 300), GSH, SOD, MDA, MPO, NF-*κ*B9(p65) DNA binding activity, mRNA expression of TNF-*α*, IL-6, HO-1, iNOS (++ both doses) Liver: expression of IL-10 (+ 150, ↑ 300) The greater the dose the more distinct the effect	Not studied

Albrahim and Binobead [30]	*Moringa oleifera* leaf water extract 200 mg/kg b.w., i.g., for 4 weeks Male albino rats, 9-10 weeks, 110-130 g	Monosodium glutamate-induced hepatotoxicity, 5 mg/kg b.w., i.g., for 4 weeks Serum: TP, globulins ↓, ALT, AST ↑ Liver: CAT, SOD, GST, GSH ↓, MDA, DNA damage, expression of PCNA and P53 proteins ↑	Serum: globulins (+), TP (++), ALT, AST (+++) Liver: CAT, SOD, GST, GSH, MDA (++); DNA damage (++), expression of PCNA and P53 proteins (++)	None

↓: a decrease vs. control; ↑: an increase vs. control; (+): a slight beneficial effect; (++): a distinct beneficial effect; (+++): a complete beneficial effect; (0): no beneficial effect.

**Table 4:** The protective effects of plant preparations against carcinogens.

Reference	Plant preparation, dose, way and time of treatment, and animals	The carcinogen, dose, way and time of exposure, and negative effects	Protective effects of plant preparation	Effects of plant *per se*
Bingül et al. [89]	*Vaccinium corymbosum* L. blueberries homogenate added to diet 8% (*w*/*w*) and given to rats for 16 weeks Male Wistar rats, 200-250 g	Diethylnitrosamine 200 mg/kg, i.p., at 4^th^, 6^th^ and 8^th^ week of the experiment Serum: ALT, AST, LDH ↑ Liver: MDA, DC, PC, GSH, GST ↑, SOD, CAT, GPx ↓ Liver: relative mRNA expression of SOD, CAT, GPx ↓, of GST-pi ↑	Serum: ALT (++), AST (+++), LDH (↓) Liver: GPx, GSH, GST, SOD, CAT (+), PC (++), MDA (+++), DC (↓) Liver: relative mRNA expression of SOD, CAT, GPx (0), of GST-pi (++)	None

Khan et al. [88]	*Phoenix dactylifera L.* (Aiwa dates) water extract 0.5 or 1.0 g/kg b.w., daily for 10 weeks, starting on the next day after diethylnitrosamine Male Wistar rats, 5-6-week-old, 100-120 g	Diethylnitrosamine-induced hepatocellular carcinoma, two doses 180 mg/kg b.w. at 15 days interval, p.o. Serum: ALT, AST, ALP, IL-1*α*, IL-1*β*, GM-CSF, MDA ↑, SOD, GR, GPx, CAT, IL-2, IL-12, IL-4 ↓	0.5 g/kg b.w.: Serum: CAT, MDA, IL-1*β* (-), IL-4 (0), IL-2 (+), SOD, GR, GM-CSF, ALP (++), ALT, AST, GPx, IL-1*α*, IL-12 (+++) 1.0 g/kg b.w.: Serum: IL-4, CAT (0), ALP, MDA, AST, IL-1*α* (↓), SOD, GR, IL-*β*, GM-CSF, IL-2 (++), ALT (+++), GPx, IL-12 (↑)	Not studied

Choi et al. [46]	*Centella asiatica* leaf (Martin Bauer GmbH & Co. KG, Vestenbergsgreuth, Germany) 75% ethanol extract 100 or 200 mg/kg, p.o., daily for 5 days following carcinogen Male Sprague-Dawley rats, 6-week-old, 180-200 g	Dimethylnitrosamine-induced liver injury 30 mg/kg, i.p. Serum: AST, ALT, ALP, total bilirubin, TNF-*α*, IL-1*β*, IL-6, INF-*γ*, IL-10, IL-12, IL-2, GM-CSF ↑ Liver: fibrosis, intralobular degeneration, focal necrosis, MDA ↑, GPx, SOD, CAT ↓	Serum: AST, INF-*γ* (↓ both doses), IL-10 (++ 100, 0 200), IL-12 (++ 100, + 200), total bilirubin, ALT (+ 100, ++ 200), ALP (++ 100, +++ 200), IL-12 (+++ 100, ++ 200), TNF-*α*, IL-1*β*, IL-6, GM-CSF (+++ both doses) Liver: SOD, CAT and fibrosis (+ 100, ++ 200), GPx (++ 100, +++ 200), intralobular degeneration and focal necrosis (++ both doses), MDA (+++ 100, ↓ 200)	Not studied

Kiziltas et al. [47]	*Ferulago angulata* flowers 80% methanol extract 150 or 300 mg/kg b.w., p.o., daily, for 21 days Male Wistar rats, 4-week-old, about 200 g	N-Nitrosodimethylamine (dimethylnitrosamine)-induced oxidative stress, 10 mg/kg b.w., i.p., for the first 7 days Liver: SOD, GPx, CAT ↓	Liver: SOD (+ 150, ++ 300), GPx, CAT (++ both doses)	Both doses: Liver: CAT ↓

Rouhollahi et al. [90]	*Curcuma purpurascens* BI. rhizome dichloromethane extract 250 or 500 mg/kg, p.o., once a day for 2 months, after azoxymethane Male Sprague-Dawley rats, 6 weeks, 200–220 g	Azoxymethane-induced aberrant crypt foci 15 mg/kg, s.c., once a week for 2 consecutive weeks Colon: CAT, GPx, SOD ↓, aberrant crypt foci formation and MDA ↑	Colon: aberrant crypt foci formation, MDA, CAT (++ both doses), GPx and SOD (++ 250, +++ 500)	Not studied

↓: a decrease vs. control; ↑: an increase vs. control; (+): a slight beneficial effect; (++): a distinct beneficial effect; (+++): a complete beneficial effect; (0): no beneficial effect; (-): intensification of the harmful effect.

**Table 5:** The protective effects of plant origin substances against toxicity of ethanol.

Reference	Plant preparation, dose, way and time of treatment, and animals	The dose, way and time of exposure, and negative effects	Protective effects of plant preparation	Effects of plant *per se*
Akbari et al. [93]	*Zingiber officinale* Roscoe (ginger) rhizome hydroalcoholic extract 1 g/kg of b.w./d, p.o., for 28 days Sprague-Dawley male rats, about 220 g	Ethanol 4 g/kg of b.w./d, p.o., for 28 days Serum: T, SHBG, DHEAs ↓ Testes: weight, Zn, Mg, Fe, Cu ↓; SOD, GPx, CAT ↓, MDA, tHcy ↑	Serum: T (++), SHBG, DHEAs (+++) Testes: Zn (-), Cu (+), Mg, weight, SOD, GPx, CAT, MDA, tHcy (+++), Fe (↑)	Serum: T ↓ Testes: Fe ↑, Zn, Mg, Cu ↓

Phunchago et al. [92]	*Tiliacora triandra* aerial parts aqueous extract, 100, 200 or 400 mg/kg b.w., p.o., for 14 days after developing ethanol dependence by15-week alcohol treatment Male Wistar rats, 8 weeks	Ethanol-dependence caused by a gradual increase of ethanol content in drinking fluid from 5% up to 30%, a 15-week alcohol treatment Hippocampus: AChE, MDA ↑, SOD, CAT, GPx ↓, neuron density in subregions CA1, CA2, CA3 and dentate gyrus ↓	Hippocampus: AChE (+ 100, ++ 200, 0 400), CAT (+++ 100 and 200, ++ 400), GPx (↑ 100 and 200, +++ 400), MDA, SOD (++ all doses), neuron density in subregions CA1, CA2, and CA3 and dentate gyrus (++ all doses)	Not studied

Liu et al. [91]	Ginseng oligopeptides extracted from roots of *Panax ginseng* C.A. Meyer (Jilin Taigu Biological Engineering Co., Ltd., China) Pretreatment: 0.0625, 0.125, 0.25, and 0.5 g/kg b.w., for 30 days, p.o., Sprague-Dawley male rats, 180-220 g	One dose of 50% ethanol 7 g (17.5 mL)/kg b.w., i.g. Serum: ALT, AST, TG, ALP, TNF-*α*, IL-6, IL-1*β* and LPS ↑, TC, HDL-c, LDL-c, TP ↓ Liver: steatosis and MDA ↑, SOD, GSH, GPx ↓	Serum: ALP (- lower doses, 0 the highest one); LDL-c, TP (0 all doses), HDL-c (0 the lowest dose, + other ones), TC (++ all doses), TG (++ lower doses, +++ higher ones), LPS (+++ except for 0.25 mg/kg ++), ALT, AST, (+++ all doses), TNF-*α*, IL-6 and IL-1*β* (↓ all doses) Liver: steatosis (++ all doses), GPx (+ lower doses, +++ 0.25, ↑ 0.5), GSH (+ lower doses, +++ higher ones), MDA (+++ all doses), SOD (+ lower doses, ++ highest one)	Not studied

Jung et al. [94]	*Glycyrrhiza uralensis* Fisher root 70% ethanol extract 100 mg/kg b.w., p.o., for 4 weeks Male C57BL/6 mice	Dietary ethanol Lieber–DeCarli liquid diet—36% of energy from ethanol, for 4 weeks Serum: TNF-*α*, ALT, AST ↑ Liver: GSH ↓, TG and mRNA expression of Srebf1, Cd36, Lpl, Fatp4 ↑	Serum: TNF-*α*, ALT, AST (++) Liver: TG, mRNA expression of Srebf1, Cd36 and LpI (++), GSH and mRNA expression of Fatp4 (+++)	Not studied

Lou et al. [65]	*Linderae radix* (prepared slices, Zhejiang Tiantaishan Wuyao Biological Engineering Co., Ltd., China) extracted from tubers of *Lindera aggregata* (Sims) Kostern, 75% ethanol extract 1, 2 or 4 g/kg, i.g., for 20 days Male Sprague-Dawley rats, 160–180 g	50% alcohol 10 mL/kg b.w., once a day for 20 days, i.g. Serum: ALT, total bilirubin, IL-6, IL-8, TNF-*α*, NF-*κ*B ↑ Portal vein: LPS ↑ Small intestine: expression of tight junction proteins claudin-1 and occludin ↓	Serum: IL-6 (+ 1 and 2, +++ 4), TNF-*α* (+++ 1, ++ 2, 0 4), NF-*κ*B (++ 1, +++ 2, and 4), ALT, total bilirubin, IL-8 (+++ all doses) Portal vein: LPS (+++ all doses) Small intestine: expression of tight junction proteins claudin-1 (+ all doses) and occludin (+++ 1, ++ 2, 0 4)	Not studied

Tang et al. [56]	*Cynara scolymus* L. (artichoke) in freeze-dried powder (Huimei Agricultural Science and Technology Co., Ltd., China) Pretreatment, 1 hour before alcohol 0.4, 0.8 or 1.6 g/kg b.w., p.o., daily for 10 days Male Institute of Cancer Research, mice, 7-week-old about 25 g	Ethanol 12 mL/kg b.w., p.o., daily for 10 days Liver index: ↑ Serum: ALT, AST, TG, TC ↑ Liver: SOD, GSH ↓, MDA↑, expression level of TLR4 and NF-*κ*B p50 ↑, score of steatosis, inflammation and necrosis ↑	Liver index: (++ all doses) Serum: ALT (0 0.4, + 0.8, +++ 1.6),TG (0 0.4, ++ 0.8 and 1.6), AST (+ 0.4 and 0.8, ++ 1.6), TC (+ 0.4, ++ 0.8 and 1.6) Liver: SOD, GSH (++ 0.4 and 0.8, +++ 1.6), MDA (+ 0.4, +++ 0.8, and 1.6), score of steatosis (+ 0.4, ++ 0.8, and 1.6), score of inflammation (+ 0.4, ++ 0.8, and 1.6), score of necrosis (++ all doses), expression of TLR4 (+ 0.4, ++ 0.8, +++ 1.6), expression of NF-*κ*B p50 (++ 0.4, +++ 0.8 and 1.6)	Not studied

Dogan and Anuk [44]	*Platanus orientalis* L. leas water extract 20 or 60 mg/mL leaf infusion *ad libitum*, for 28 days Male Wistar rats, 2 months aged, about 200 g	20% ethanol water *ad libitum* for 28 days Serum: AST, ALT, LDH, GGT, UA, urea ↑, cholesterol, HDL-c ↓ Liver: GSH ↓, GST, MDA ↑ Erythrocytes: GST, MDA ↑ Kidney: MDA ↑, SOD, GPx, CAT ↓	Serum: GGT (- 20, ++ 60), ALT, urea (+ 20, 0 60), HDL-c (+ both doses), LDH (++ 20, +++ 60), cholesterol (++ 20, + 60), AST, UA (+++ both doses) Liver: GSH and GST (+ both doses), MDA (++ both doses) Erythrocytes: GST (0 both doses), MDA (++ 20, +++ 60) Kidney: SOD (- 20, + 60), CAT (- 20, ++ 60), MDA (+++ both doses), GPx (+++ 20, ↑ 60)	Not studied

↓: a decrease vs. control; ↑: an increase vs. control; (+): a slight beneficial effect; (++): a distinct beneficial effect; (+++): a complete beneficial effect; (0): no beneficial effect; (-): intensification of the harmful effect.

**Table 6:** The protective effects of plant substances against toxicity of antipyretic and analgesic drugs.

Reference	Plant preparation, dose, way and time of treatment, and animals	The name of drug, dose, way and time of exposure, and negative effects	Protective effects of plant preparation	Effects of plant *per se*
Bouzenna et al. [21]	*Pinus halepensis* L. needles essential oil Pretreatment, 1% (*w*/*w*), with a dose of 1 mL/kg, p.o., for 56 days Female Wistar rats, 150-200 g	Aspirin-induced liver and kidney damage 600 mg/kg thrice a day for 4 days, p.o. Weight: body, liver, kidney ↓ Serum: glucose, TC, AST, ALT, LDH, CR, urea ↑ Liver: TBARS ↑, SOD, CAT, GPx ↓ Kidney: TBARS ↑, SOD, CAT, GPx ↓	Weight: body, liver, kidney (+++) Serum: glucose, TC, AST, ALT, LDH, CR, urea (+++) Liver: TBARS, SOD, CAT, GPx (+++) Kidney: TBARS, SOD, CAT, GPx (+++)	None

Ahmad and Zeb [18]	*Trifolium repens* leaf water extract 11 mg of solid weight/mL; 1, 2, or 3 mL of extract per day for 2 weeks Male albino mice about 28.6 g	Acetaminophen-induced hepatotoxicity 300 mg/kg for 2 weeks Serum: TC, TG, LDL-c, ALT, AST, ALP, glucose ↑, HDL-c ↓ Blood: RBC, HGB, HCT, PLT ↓, WBC ↑ Liver: TBARS ↑, GSH ↓	1 mL: Serum: ALP, glucose (0), TC, TG (+), HDL-c, ALT, AST (++), LDL-c (+++) Blood: PLT (0), HCT (+), WBC (++), RBC, HGB (+++) Liver: GSH (+), TBARS (++) 2 mL: Serum: TC (+), ALT, AST, ALP, TG, HDL-c, glucose (++), LDL-cholesterol (+++) Blood: PLT (+), RBC, HGB, HCT, WBC (+++) Liver: GSH, TBARS (++) 3 mL: Serum: HDL-c (0), TC, AST, ALT, AST (++), glucose, LDL-c (+++) Blood: PLT, RBC, HGB, HCT, WBC (+++) Liver: TBARS, GSH (++)	Studied only for the 1 mL dose Serum: TC ↓, HDL-c ↑

Azim et al. [19]	*Moringa peregrina* leaves 70% ethanol extract 200 mg/kg b.w. p.o., one hour prior to acetaminophen, for 4 weeks Female albino rats, about 160 g	Acetaminophen-induced hepatotoxicity 750 mg/kg b.w., p.o. Serum: ALT, AST, ALP, GGT ↑ Blood: GSH, CAT, SOD ↓, MDA ↑ Liver: GSH, CAT, SOD ↓, GPx, MDA ↑ Brain: GSH, SOD ↓, GPx and MDA ↑	Serum: ALT, ALP (++), AST, GGT (+++) Blood: GSH, CAT, SOD, MDA (+++) Liver: GSH, CAT, SOD, GPx, MDA (+++) Brain: GPx, MDA (++), GSH, SOD (+++)	Not studied

Mishra et al. [97]	*Pandanus odoratissimus* root ethanolic extract 200 or 400 mg/kg b.w., p.o., once a day for 7 days Wistar rats 180-200 g	Paracetamol-induced hepatotoxicity 2 g/kg b.w., p.o., on the 5^th^ day Serum: AST, ALT, ALP, direct bilirubin, total bilirubin, TG ↑	Serum: ALT, AST, ALP, direct bilirubin, total bilirubin, TG (++); The protective effect was better for the higher dose.	Not studied

Rashid et al. [40]	*Fagonia olivieri* DC. aerial parts 95% methanolic extract 200 and 400 mg/kg, i.g., for 7 days Male Sprague-Dawley rats, 6-week-old, 180-200 g	Acetaminophen-induced toxicity 750 mg/kg for 7 days, i.g. Serum: AST, ALT, ALP, LDH, total bilirubin, cholesterol, TG, HDL, LDL ↑ Blood: HGB, WBC ↓, PLT ↑ Liver: weight, CAT, SOD, GPx, GR, GSH and TP ↓, DNA fragmentation and TBARS ↑	Serum: AST, ALT, LDH, LDL (++) both doses, ALP, total bilirubin, TG, HDL (++ 200, +++ 400), cholesterol (+++ 200, 400 ↓) Blood: HGB (++ both doses), WBC (+++ both doses), PLT (+++ 200, ↑ 400) Liver: TP (0 200, ++ 400), DNA fragmentation, SOD, GR, GSH, TBARS (++ both doses), weight, CAT, GPx (++ 200, +++ 400)	400 mg/kg studied only Serum: TG, HDL, cholesterol ↓ Blood: WBC ↑, HGB ↓ Liver: TBARS ↓

Ebada [95]	*Chamomilla recutita* L. (chamomile) and *Cuminum cyminum* L. (green cumin) essential oils (Hashem Brothers for Essential Oils and Aromatic products) Pretreatment p.o., for 14 days, 400 mg/kg/day (cumin), 250 mg/kg/day (chamomile) Male Wistar rats, 180-200 g	Acetaminophen-induced hepatotoxicity 1 g/kg p.o., a single dose on the 14^th^ day Serum: ALT and AST ↑ Liver: GSH ↓, MDA and SOD ↑ Liver histopathology: hepatocyte necrosis, inflammation, degeneration, dilated central vein, hemorrhage ↑	Cumin essential oil: Serum: ALT and AST (+++) Liver: GSH (0), MDA (-) and SOD (0) Liver histopathology: hepatocyte necrosis, inflammation, degeneration, dilated central vein, hemorrhage (++) Chamomile essential oil: Serum: ALT (++) and AST (0) Liver: MDA (-), GSH, SOD (++) Liver histopathology: hepatocyte necrosis, hemorrhage inflammation, dilated central vein, degeneration (+)	Not studied

Baali et al. [20]	Rich-polyphenol (n-butanol) fractions of 80% methanolic extract of *Genista quadriflora* Munby and 70% methanolic extract of *Teucrium polium geyrii* Maire Pretreatment 300 mg/kg daily, p.o., for 10 days Male Wistar rats, about 142 g	Acetaminophen-induced hepatotoxicity A single dose of 1 g/kg, p.o. Plasma: AST, ALT ↑ Liver: SOD, GPx, GR, GSH, GST ↓, CYP2E1, TBARS and mRNA of TNF-*α* expression ↑ Liver mitochondria: CS and respiratory chain complexes I and II ↓	*Genista quadriflora* Plasma: AST, ALT (+++) Liver: SOD, GPx, GSH, GST, TBARS, CYP2E1 and mRNA of TNF-*α* expression (+++), GR ↑ Liver mitochondria: CS and respiratory chain complex I (+++), respiratory chain complex II ↑ *Teucrium polium geyrii* Plasma: AST, ALT (+++) Liver: SOD, GPx, GSH, GST, TBARS, CYP2E1 and mRNA of TNF-*α* expression (+++), GR ↑ Liver mitochondria: CS (+++), respiratory chain complexes I and II ↑	Not studied

Uthaya Kumar et al. [96]	*Cassia surattensis* seed methanol extract Pretreatment 250 or 500 mg/kg b.w., p.o., once daily for 7 days Male Swiss albino mice, 6-8-week-old, 25-30 g	Paracetamol-induced hepatotoxicity A single dose of 1 g/kg b.w., p.o. Relative liver weight: ↑ Serum: ALT, AST, ALP ↑ Liver: GSH, SOD ↓, MDA ↑	Relative liver weight: (+++ both doses) Serum: ALT, AST, ALP (++ both doses) Liver: MDA and GSH (++ 250, +++ 500), SOD (++ 250, ↑ 500)	Not studied

Chinnappan et al. [22]	*Eurycoma longifolia* root water extract Physta® (Biotropics Malaysia) 100, 200 or 400 mg/kg, p.o., 1 hour before paracetamol, for 14 days Male Wistar rats, 12-week-old, 120-150 g	Paracetamol-induced nephrotoxicity 200 mg/kg daily for 14 days, i.p. Serum: TP, ALB ↓, creatinine ↑ Blood: urea ↑ Creatinine clearance: ↓	100 mg/kg: Serum: ALB, creatinine (0), TP (+) Blood: urea and (0) Creatinine clearance: (0) 200 and 400 mg/kg: Serum: TP, ALB, creatinine (+++) Blood: urea (+++) Creatinine clearance: (+++)	Not studied

↓: a decrease vs. control; ↑: an increase vs. control; (+): a slight beneficial effect; (++): a distinct beneficial effect; (+++): a complete beneficial effect; (0): no beneficial effect; (-): intensification of the harmful effect.

**Table 7:** The protective properties of plant extracts against side effects of antibiotics.

Reference	Plant preparation, dose, treatment way and time, and animals	The name of drug, dose, exposure way and time, and negative effects	Protective effects of plant preparation	Effects of plant *per se*
Valipour et al. [98]	*Ferulago angulata* Ethanol : water (70 : 30, *v*/*v*) extract 200, 400, or 800 mg/kg b.w., p.o., for 7 days Male Wistar rats, about 200 g	Gentamicin 120 mg/kg b.w., daily for 7 consecutive days, i.p. Serum: HDL ↓; TG, TC, LDL, UA, urea, CR, PC, TNF-*α*, MDA ↑ Kidney: CAT, SOD and AA ↓, MDA and relative expression of TNF-*α* ↑	Serum: HDL (0 200 and 400, +++ 800), CR (+ 200, ++ 400 and 800), PC (+ 200, ++ 400, +++ 800), UA and urea (++ all doses), TC, LDL, TNF-*α* and MDA (++ 200 and 400, +++ 800), TG (++ 200, +++ 400 and 800) Kidney: SOD (0 200, ++ 400 and 800), relative expression of TNF-*α* (0 200, ++ 400, +++ 800), AA (0 200, +++ 400 and 800), CAT (++ 200 and 400, +++ 800), MDA (++ 200, +++ 400 and 800)	Not studied

Boroushaki et al. [29]	*Rheum turkestanicum* root 70% ethanol extract 100 or 200 mg/kg, for 6 days, i.p., 1 hour before gentamycin Male Wistar rats, 250-300 g	Gentamycin 80 mg/kg/day, for 6 days, i.p. Serum: CR and urea ↑ Kidney: MDA ↑, total SH content ↓ Urine: protein and glucose ↑	Serum: CR, urea (++ both doses) Kidney: MDA, total SH content (++ both doses) Urine: protein (0 100, ++ 200), glucose (+ 100, ++200)	Not studied

Apaydin Yildirim et al. [28]	*Helichrysum plicatum* DC. subsp. *plicatum* aerial parts ethanol extract 100 or 200 mg/(kg·d) i.p., for 8 days Male Sprague-Dawley rats	Gentamicin 80 mg/(kg·d) i.p., for 8 days Serum: BUN and creatinine ↑ Liver: CAT and SOD ↓; MDA, degeneration, necrosis, inflammatory cells, biliary hyperplasia ↑ Kidney: SOD, CAT, GPx ↓, MDA, inflammatory cells, degeneration, necrosis ↑	Serum: BUN and creatinine (++ both doses) Liver: SOD (++ 100, 0 200), MDA (++ 100, +++ 200), CAT (+++ 100, ++ 200), necrosis, degeneration (++ 100, + 200), inflammatory cells (+++ 100, + 200), biliary hyperplasia (++ both doses) Kidney: GPx (both doses), SOD (+++ 100, 0 200), MDA, CAT (+++ 100, ++ 200) Kidney: degeneration and inflammatory cells (++ 100, + 200), necrosis (++ both doses)	Serum: BUN (↓100, ↑ 200) Liver: CAT (↓ 200), inflammatory cells, biliary hyperplasia and necrosis (↑ 200), degeneration (↑ both) Kidney: CAT (↑ both doses), inflammatory cells and necrosis (↑ 200), degeneration (↑ both)

Zhang et al. [99]	*Panax notoginseng* saponins (Guangxi Wuzhou Pharmaceutical Co., Ltd. Hangzhou, China) 10 mg/kg, twice a day for 14 days, i.m. Female ICR mice, 18-20 g	Polymyxin-induced nephrotoxicity 15 mg/kg twice a day for 14 days, i.m. Kidney weight: body weight ratio: ↑ Serum: BUN, CR ↑ Kidney: SOD ↓, MDA and number of apoptotic cells ↑	Kidney weight: body weight ratio: (++) Serum: BUN, CR (+++) Kidney: number of apoptotic cells (++), SOD and MDA (+++)	None

↓: a decrease vs. control; ↑: an increase vs. control; (+): a slight beneficial effect; (++): a distinct beneficial effect; (+++): a complete beneficial effect; (0): no beneficial effect; (-): intensification of the harmful effect.

**Table 8:** The protective effects of plant preparations against side effects of anticancer agents.

Reference	Plant preparation, dose, treatment way and time, and animals	The name of drug, dose, exposure way and time, and negative effects	Protective effects of plant preparation	Effects of plant *per se*
Kim et al. [100]	*Dendropanax morbifera* The CHCl_3_ fraction of 70% methanol leaf extract 25 mg/kg once at 24 h before cisplatin and once a day for 5 days after, i.p. Sprague Dawley male rats, 8 weeks, about 200 g	Cisplatin, a single administration of 6 mg/kg, i.p. Body weight: ↓ Kidney/body weight ratio: ↑ Serum: BUN, CR ↑ Kidney: SOD ↓, cleaved caspase-3 and acute tubular necrosis score ↑	Body weight: (++) Kidney/body weight ratio: (+++) Serum: CR (++), BUN (+++) Kidney: acute tubular necrosis score (++), SOD and cleaved caspase-3 (+++)	Not studied

Kpemissi et al. [102]	*Combretum micranthum* leaf ethanol-water (8 : 2) extract 200 or 400 mg/kg/day p.o., for 10 days Male Wistar rats, 6-8-week-aged, 200-250 g	Cisplatin 7.5 mg/kg, i.p., a single injection on the 5^th^ day Body weight: ↓ Relative kidney weight: ↑ Serum: CR, urea, UA, ALT, AST, GGT, ALP ↑, TP, ALB, Ca, Mg, P, NO, FRAP ↓ Urine: CR, urea, UA, P ↓, TP, ALB, Ca, Mg, ↑ Kidney: MDA ↑, FRAP, NO, GSH ↓	Body weight (++ both doses) Relative kidney weight: (+++ 200, ++ 400) Serum: urea (+++ 200, ++ 400), CR, UA TP, ALB, Ca, Mg, P, ALT, AST, ALP, NO, FRAP (+++ both doses), GGT (↓ both doses) Urine: CR, urea, UA TP, (+++ 200, ++ 400), ALB, Ca, Mg, P (+++ both doses) Kidney: NO, GSH (++ both doses), FRAP (+++ 200, ++ 400), MDA (+++ both doses)	Not studied

Hosseinian et al. [16]	*Nigella sativa* seeds 70% ethanol extract 100 or 200 mg/kg, i.p. (i) Pretreatment (6 days)+saline for 5 days after cisplatin (ii) Pretreatment (6 days)+extract for 5 days after cisplatin Male Wistar rats, 230-300 g	Cisplatin 6 mg/kg on the 6^th^ day of the experiment, i.p. Serum: total SH ↓, MDA ↑ Kidney: total SH ↓, MDA ↑ slightly Renal tissue damage: ↑	(i) Serum: MDA (+++ both doses), total SH (+++100, ↑ 200) Kidney: MDA (- both doses), total SH (+++ both) Renal tissue damage: (++ both doses) (ii) Serum: MDA (+++ both doses), total SH (↑ both) Kidney: MDA (- 100, +++ 200), total SH (+++ both) Renal tissue damage: (++ both doses)	Not studied

Chen et al. [25]	Total flavonoids from *Clinopodium chinense* (Benth.) Pretreatment 20, 40, or 80 mg/kg, i.g., for 15 days Male Sprague-Dawley rats, 220-250 g	Doxorubicin-induced cardiotoxicity 3 mg/kg, i.p., every two days for a total of three injections Body and heart weight: ↓ Serum: AST, LDH, CK ↑ Heart: SOD, CAT, GPx ↓, MDA ↑	Body and heart weight: (++ 20, +++ 40 and 80) Serum: CK, LDH (++ 20, +++ 40 and 80), AST (+++ all doses) Heart: GPx (+ 20, ++ 40 and 80), CAT (++ all doses), MDA, SOD (++ 20, +++ 40 and 80)	80 mg/kg only studied: none

Ahmed et al. [24]	*Allium sativum* (garlic) aqueous extract 1 mL/100 g b.w., p.o., daily for 7 days before and 7 days after methotrexate Male Wistar rats, 100–120 g	Methotrexate-induced nephrotoxicity 20 mg/kg, a single injection, i.p. Serum: urea, CR, K, P ↑, Na ↓ Kidney: GSH, CAT ↓, MDA, ADA, NO ↑	Serum: urea (↓), P (+), CR, K, Na (+++) Kidney: MDA, NO (++), GSH, CAT, ADA (+++)	None

Tag et al. [37]	*Morus nigra* (mulberry) leaves 50% ethanol extract 500 mg/kg, i.g., daily for 14 days Male albino rats, 180–200 g	Methotrexate-induced hepatotoxicity, a single dose, 20 mg/kg, on 4^th^ day, i.p. Liver weight/body weight: ↑ Serum: AST, ALT, ALP, LDH ↑ Liver: total pathological score ↑, mean score of hepatocyte degeneration, congestion, cellular infiltration, fibrosis ↑	Liver weight/body weight: (++) Serum: ALP (++), AST, ALT, LDH ↓ Liver: total pathological score (++), mean score of hepatocyte degeneration, congestion, cellular infiltration and fibrosis (++)	Serum: AST, ALT, ALP, LDH ↓

Moghadam et al. [103]	*Curcuma longa* L., ethanol extract 100 or 200 mg/kg, p.o., for 30 days Male Wistar rats, 220–280 g	Methotrexate-induced hepatotoxicity, a single dose 20 mg/kg i.p., on day 30^th^ Body weight: Liver weight: ↑ Serum: ALB, TP ↓, ALT, AST, ALP, bilirubin ↑ Plasma: TAS ↓ Liver: SOD, GPx, CAT ↓, MDA, number of neutrophils, degree of injury ↑	Body weight: Liver weight ratio: (++ both doses) Serum: AST, ALP (++ both doses), ALB, ALT, bilirubin, TP (++ 100, +++ 200) Plasma: TAS (++100, +++200) Liver: MDA, number of neutrophils (+ 100, ++ 200), degree of injury (++ both doses), SOD, CAT, GPx (++ 100, +++ 200)	The higher dose: Liver: CAT ↑ Plasma: TAS ↑

Omole et al. [26]	Kolaviron—a mixture of flavonoids obtained from *Garcinia kola* seeds Pretreatment 200 or 400 mg/kg/d, p.o., for 14 days Male Wistar rats, 120-150 g	Cyclophosphamide 50 mg/kg/d, i.p., 24 hours after the last dose of kolaviron, for 3 days Relative heart weight: ↑ Heart: SOD, CAT, GPx, GSH ↓, LDH, CK, MDA, H_2_O_2_, MPO and cTn I ↑	Relative heart weight: (+++ 200, ++ 400) Heart: LDH, CK, cTn I, MPO, and GSH (++ both doses), GPx, CAT, MDA, H_2_O_2_ (+++ 200, ++ 400), SOD (+++) both doses	None

Sheweita et al. [101]	Essential oils of *Foeniculum vulgare* Miller (fennel) seeds, *Cuminum cyminum* L. (cumin) seeds, and *Syzygium aromaticum* L. (clove) flower buds (0.12 mL/kg b.w., 0.10 mL/kg b.w., and 0.106 mL/kg b.w., (1/50 LD_50_ doses), respectively), p.o. for 28 days Male Swiss albino mice, about 25 g	Cyclophosphamide 2.5 mg/kg b.w. for 28 days, p.o. Serum: ALT, AST, ALP ↑ Liver (S9 fraction): TBARS ↑; GSH, GPx, GR, GST, SOD, CAT ↓ Hepatic microsomal fraction: NADPH-cytochrome c reductase ↑	Serum: ALT, AST, ALP (++ all oils) Liver (S9 fraction): TBARS (++ clove oil, +++ fennel and cumin oils); GPx, GSH, GR, SOD, CAT (+++ all oils); GST ↑ all oils Hepatic microsomal fraction: NADPH-cytochrome c reductase (0 all oils)	Liver: TBARS ↓, GPx, CAT, GST ↑ (all oils), GR ↑ (cumin and clove), GSH, SOD ↑ (cumin), NADPH-cytochrome c reductase ↑ (all)

Rahate and Rajasekaran [27]	*Desmostachya bipinnata* Stapf (L.) roots—the polyphenolic fraction of 70% methanol *extract* 100 or 200 mg/kg b.w., p.o., 21 days Sprague-Dawley female rats, 150–200 g	Tamoxifen citrate 45 mg/kg b.w., p.o., 10^th^–21^st^ days Serum: protein ↓, AST, ALT, ALP, cholesterol, TG, urea, CR, UA, bilirubin ↑ Liver: LPO↑, GSH, GPx, SOD, CAT ↓	Serum: protein, AST, ALT, cholesterol, TG, urea, UA (++ both doses), CR, bilirubin (++ 100, +++ 200), ALP (++ 100, ↓ 200) Liver: CAT (+ 100, ++ 200), LPO, GSH, GPx, SOD, (++ both doses)	Not studied

↓: a decrease vs. control; ↑: an increase vs. control; (+): a slight beneficial effect; (++): a distinct beneficial effect; (+++): a complete beneficial effect; (0): no beneficial effect; (-): intensification of the harmful effect.

**Table 9:** The protective effects of plant preparations against side effects of various drugs.

Reference	Plant preparation, dose, treatment way and time, animals	The name of drug, dose, exposure way and time, negative effects	Protective effects of plant preparation	Effects of plant *per se*
Ben Saad et al. [104]	*Opuntia ficus-indica* cladode extract obtained by homogenization with 10 mM Tris HCl, pH 7.4, and subsequent centrifugation Pretreatment, 100 mg/kg b.w. 30-day pretreatment and then for 30 days together with lithium Male Wistar rats, 2 months	Li (lithium carbonate), 25 mg/kg b.w., i.p., twice daily for 30 days Serum: glucose, TC, TG, ALT, AST, LDH, ALP ↑ Blood: RBC, HGB, HCT ↓, WBC ↑ Liver: SOD, CAT, GPx ↓, MDA ↑	Serum: ALT, AST (++), glucose, TC, TG, ALP, LDH (+++) Blood: RBC, HGB, HCT, WBC (++) Liver: CAT (++), SOD, GPx, MDA (+++)	None

Khalil et al. [105]	*Withania somnifera* leaf 70% ethanol extract Pretreatment, 100 mg/kg b.w., for 4 weeks Male Wistar rats, 140–160 g	Isoproterenol 85 mg/kg b.w., s.c. on 29^th^ and 30^th^ days Serum: TC, TG, VLDL-c, cTnI, CK-MB, LDH, AST, ALT ↑, HDL-c ↓ Heart: SOD, GR, GPx, GST ↓, MDA, weight ↑	Serum: CK-MB (0), TC, TG, VLDL-c, cTnI, LDH (++), HDL-c, AST, ALT (+++) Heart: SOD, weight (++), GR, GPx, GST, MDA (+++)	Serum: HDL-c ↑ Heart: GPx, GST ↑

Dianita et al. [106]	*Labisia pumila* var. *alata* water and 80% ethanolic extracts 100, 200, or 400 mg/kg, p.o., for 28 days Male Wistar rats, 150-200 g	Isoproterenol, 85 mg/kg of s.c., on 29^th^ and 30^th^ day of the experiment Serum: cTnI, CK-MB, LDH, AST, ALT ↑, SOD, CAT, GPx ↓ Heart: SOD, CAT, GPx ↓	Serum: cTnI (water extract ++ all doses, ethanol extract ++ 100 and 200, +++ 400), CK-MB, LDH, AST, ALT, SOD, CAT, GPx (++ both extracts all doses) Heart: SOD, GPx (water extract + 100, ++200 and 400, ethanol extract ++ all doses), CAT (++ both extracts, all doses)	Not studied

Shahat et al. [107]	*Rhus tripartita* 80% methanol extract of the stem part of male genotype 250 or 500 mg/kg/day p.o., for 21 days (19-day-pretreatment) Male Wistar rats, about 180 g	Isoproterenol 85 mg/kg, s.c. on 20^th^ and 21^st^ days Serum: AST, ALT, GGT, ALP, LDH, CK, TC, TG, LDL-c, VLDL-c↑, HDL-c ↓ Heart: MDA ↑, NP-SH and TP ↓	Serum: LDH, HDL-c (+ 250, ++500), AST, ALT, GGT, ALP, CK, TC, TG, LDL-c and VLDL-c (++ both doses) Heart: MDA, NP-SH and TP (++ both doses)	Not studied

Kemka Nguimatio et al. [108]	*Aframomum melegueta* seed water and methanol extracts 20 or 100 mg/kg for 28 days Male Wistar rats, sexually experienced, 3-month-old, 200-250 g	Propylthiouracil-induced hypothyroidism causing ejaculatory complications 10 mg/[kg·day] for 28 days Plasma: T ↓, TSH and prolactin ↑ Proejaculatory activity (contractile activity of the striated bulbospongiosus muscles): number of contractions after tactile stimulation and pharmacological activation (dopamine) ↓, intraseminal pressure after tactile stimulation and pharmacological activation (dopamine) ↓	Water extract: Plasma: TSH (0 both doses), T (+ 20, +++ 100), prolactin (+ 20, ++ 100) Proejaculatory activity: contractions number after both stimuli (+++ 20, ↑ 100), intraseminal pressure: tactile stimulation (↑ both doses), pharmacological activation (+++ both doses) Methanol extract: Plasma: TSH (0 20, +100), T (+ 20, ++ 100), prolactin (++ 20, +100) Proejaculatory activity: contractions number after both stimuli (↑ both doses), intraseminal pressure: tactile stimulation (↑ both doses), pharmacological activation (+++ 20, ↑100)	Not studied

Nasri et al. [109]	Green tea 70% ethanol extract Posttreatment: after iodixanol, 10 mg/kg/d, i.p., for 3 days Pretreatment 10 mg/kg/d, i.p., for 3 days and iodixanol on the 3^rd^ day Male Wistar rats, 6-week-aged, 200-250 g	Iodixanol, a contrast medium 10 mL/kg, i.v., a single dose Renal tubular cells: dilatation, degeneration, vacuolization, debris ↑	Posttreatment: Renal tubular cells: dilatation, degeneration, vacuolization, debris (++) Pretreatment: Renal tubular cells: dilatation, degeneration, vacuolization, debris (++) Pretreatment showed a slightly better effect	Not studied

El-Rahman et al. [110]	*Saussurea lappa* roots 70% ethanol extract 600 mg/kg, p.o., daily Cotreatment for 2 weeks concurrently with the drug Pretreatment for 1 week and then for 2 weeks concurrently with the drug Male albino rats,150–160 g	Triamcinolone acetonide 40 mg/kg, i.p., twice a week for 2 weeks Serum: TNF-*α*, CRP, IL-12, IgG, IgM ↓ Lung: GPx, SOD ↓, MDA ↑ Spleen: GPx, SOD ↓, MDA ↑ Histopathological lesion score: spleen and lung ↑	Cotreatment: Serum: TNF-*α* and CRP (0), IL-12 and IgG (+), IgM (++) Lung: GPx (+), SOD, MDA (++) Spleen: GPx, SOD, MDA (++) Histopathological lesion: spleen and lung (++) Pretreatment: Serum: TNF-*α*, IL-12, CRP (0), IgG, IgM (++) Lung: GPx, SOD, MDA (++) Spleen: GPx, SOD (++), MDA (+++) Histopathological lesion: spleen and lung (++)	Serum: TNF-*α*, CRP, IL-12, ↓, IgG, IgM ↑ Lung: GPx, SOD ↑, MDA ↓ Spleen: GPx, SOD ↑, MDA ↓

↓: a decrease vs. control; ↑: an increase vs. control; (+): a slight beneficial effect; (++): a distinct beneficial effect; (+++): a complete beneficial effect; (0): no beneficial effect.

**Table 10:** The protective effects of plant origin materials in the animal model of arthritis.

Reference	Plant extract, dose, way and time of treatment, and animals	Inducing factor and negative effects	Protective effects of plant material	Effects of plant *per se*
Choudhary et al. [49]	*Spinacia oleracea* leaf ethanol extract 250 or 500 mg/kg/day, for 28 days, p.o. Healthy and pathogen-free female Sprague-Dawley rats, 4–16 weeks, 180-200 g	Osteoarthritis, induced by monosodium iodoacetate, a single injection of 3 mg through the intra-articular joint of the left knee Serum: GST, COMP ↑ Urine: CTX-II ↑ Isolated bone region containing cartilage and sub-chondral bone: relative mRNA expression of IL-1*β*, TNF-*α*, COL10, MMP-1, MMP-3, MMP-13 ↑, relative mRNA expression of BMP2, COL2, AGGRECAN, TIMP1, TIMP2 ↓	Serum: COMP (+ 250, ++ 500), GST (++ both doses) Urine: CTX-II (+ 250, ++ 500) Isolated bone region containing cartilage and sub-chondral bone: relative mRNA expression of TNF-*α* and MMP-13 (+ 250, ++ 500); relative mRNA expression of IL-1*β*, COL10, MMP-1, MMP-3 (++ both doses); relative mRNA expression of BMP2, COL2 and TIMP1 (0 250, ++ 500), relative mRNA expression of TIMP2 and AGGRECAN (++ both doses)	Not studied

Jeong et al. [48]	*Morus alba* L. leaf water extract 100 mg/kg, p.o., once per day for 3 weeks Male Sprague Dawley rats, 180-240 g, 5-week-old	Osteoarthritis, induced by monosodium iodoacetate, a single injection 3 mg/kg into the right knee joint Serum: IL-1*β*, IL-6, TNF-*α*, INF-*γ*, NO, PGE_2_, COMP, CTX-II ↑ Articular cartilage: expression of MMP-13 ↑	Serum: INF-*γ* (0), IL-1*β*, IL-6, TNF-*α*, NO, PGE_2_, CTX-II, COMP (++) Articular cartilage: expression of MMP-13 (++)	Articular cartilage: a slight increase in MMP-13 expression

Sundaram et al. [55]	Guggulipid, an ethyl acetate extract rich in lipids from *Commiphora whighitii* gum resin, obtained by extraction of guggulu oleoresin (NKCA pharmacy, Musurum India) 50 or 100 mg/kg/day, for 15 days, p.o. Adult Wistar rats, 10-week-old	Experimental arthritis induced by 100 *μ*L of FCA (Freund's complete adjuvant containing 10 mg/mL heat-killed *Mycobacterium tuberculosis*) injected at the back surface of right hind paw, s.c. Blood: WBC ↑, RBC, HGB, PLT ↓ Serum: expression of MMP-1, MMP-3, MMP-9, MMP-13 ↑ Serum: ALT AST, SOD, CAT and GST ↑ Liver and spleen: ROS, hydroperoxides, LPO, PC, NO_2_^−^, GSSG, SOD, CAT, GST ↑, GSH, GPx, GR ↓ Ankle joint homogenate: ACP, ALP ↑	Blood: HGB (0 50, ++ 100), WBC, RBC (++ both doses), PLT (++ 50, +++ 100) Serum: MMP-1 (+ 50, +++ 100), MMP-3 (+ 50, ++ 100), MMP-9 (++ 50, +++ 100), MMP-13 (+++ both doses) Serum: ALT, AST, CAT (++ 50, +++ 100), SOD and GST (+++ both doses) Liver: ROS, hydroperoxides, GPx, CAT (++ both doses), LPO, PC, NO_2_^−^, SOD, GSH, GSSG and GR (++ 50, +++ 100) Spleen: ROS, hydroperoxides (++ both doses), LPO, PC, NO_2_^−^, GR, GSH, GSSG (++ 50, +++ 100), SOD, CAT, GST (+++ both doses) Ankle joint homogenate: ACP (+ 50, ++ 100), ALP (++ both doses)	Not studied

↓: a decrease vs. control; ↑: an increase vs. control; (+): a slight beneficial effect; (++): a distinct beneficial effect; (+++): a complete beneficial effect; (0): no beneficial effect.

**Table 11:** The protective effects of plant origin materials in the animal model of neurodegenerative disorders.

Reference	Plant extract, dose, way and time of treatment, and animals	Disorder, inducing factor, and negative effects	Protective effects of plant material	Effects of plant *per se*
Hritcu et al. [32]	*Piper nigrum* fruits methanolic extract 50 or 100 mg/kg, p.o., daily, for 21 days Male Wistar rats, 3 month-old, about 250 g	Animal model of Alzheimer's disease 400 pmol A*β* (1–42), i.c.v., 20 days prior to methanolic extract Amygdala: GPx, GSH ↓, CAT, PC, MDA ↑	The lower dose: Bilateral amygdala: GPx ↑, GSH (0), PC and CAT ↓, MDA (+++) The higher dose: MDA, PC, CAT ↓, GSH (++), GPx (+++)	Not studied

Malik et al. [39]	*Celastrus paniculatus* seed ethanol extract Pretreatment for 6 days 100 or 200 mg/kg, p.o., and then treatment concomitantly with 3-nitropropionic acid Male Wistar rats, 220–250 g	3-Nitropropionic acid-induced Huntington's disease symptoms, 10 mg/kg, i.p., for 14 days Body weight ↓ Striatum: MDA, NO_2_^−^ ↑, CAT, SOD, GSH ↓ Cortex: MDA, NO_2_^−^ ↑, CAT, SOD, GSH ↓	Body weight: (++ both doses) Striatum: MDA, NO_2_^−^, CAT, SOD, GSH (++ both doses) Cortex: MDA, NO_2_^−^, CAT, SOD, GSH (++ both doses)	Not studied

Chonpathompikunlert et al. [31]	*Apium graveolens* L., 70% methanolic extract 125, 250, and 375 mg/kg b.w., p.o., for 21 days Male C57BL/6 mice, 2 months, 25–30 g	Parkinson's disease induced by 1-methyl-4-phenyl-1,2,3,6-tetrahydropyridine, 15 mg/kg per day, i.p., divided into 4 injections at 2 h intervals on a single day Cortex: MDA, MAO-A, MAO-B ↑, GPx ↓ Striatum: MDA, MAO-A, MAO-B ↑, GPx ↓	The lowest dose: Cortex and striatum: MAO-A, GPx (0), MDA, MAO-B (++) The middle dose: Cortex: MAO-B MAO-A, GPx (++), MDA (+++) Striatum: MAO-B MAO-A, GPx and MDA (++) The highest dose: Cortex: MDA, MAO-B, MAO-A and GPx (+++) Striatum: MDA ↓, MAO-B, MAO-A, GPx (+++)	Not studied

Wang et al. [38]	*Rhizoma drynariae* (a commercial product) made from dried roots of *Drynaria fortunei* water extract 20 mg/kg p.o., daily, for 14 or 28 days Male Sprague-Dawley rats, about 250 g	Controlled cortical impact (CCI) model of traumatic brain injury Plasma: IL-6 after 1, 3, 7, 14, 21, and 28 days ↑ Brain: microglial activation after 1, 3, 7, and 14 days ↑	Plasma: IL-6 (0 after 3 and 7 days, ++ after 1 day, +++ after 14, 21, and 28 days) Brain: microglial activation (0 after 1 day, ++ after 3, 7, and 14 days)	Not studied

Xu et al. [60]	Rhubarb (three species are assigned as official Rhubarb, i.e., *Rheum palmatum* L., *Rheum tanguticum* Maxim. ex Balf., and *Rheum officinale* Baill), a commercial product, water extract 3, 6, 12 g/kg of Rhubarb p.o., after trauma Male Sprague-Dawley rats, 200-300 g sacrificed 8, 16, or 24 hours after Rhubarb	Controlled cortical impact (CCI) model of traumatic brain injury Brain 8 h after Rhubarb: MDA ↑, SOD, CAT, GSH/GSSG ↓ Brain 16 h after Rhubarb: MDA ↑, SOD, CAT, GSH/GSSG ↓ Brain 24 h after Rhubarb: MDA ↑, SOD, CAT, GSH/GSSG ↓	Brain 8 h after Rhubarb: MDA and CAT (0 3 and 6, ++ 12), SOD (0 3, + 6, ++ 12), GSH/GSSG (+3, ++ 6, +++12) Brain 16 h after Rhubarb: MDA (0 3, ++ 6 and 12), SOD (++3 and 6, +++ 12), CAT (0 3, ++ 6 and 12), GSH/GSSG (0 3, + 6, ++12) Brain 24 h after Rhubarb: MDA (++ 3 and 6, +++ 12), SOD (+ 3, ++ 6, +++ 12), CAT (0 3, ++ 6 and 12), GSH/GSSG (+ 3, ++ 6, +++ 12)	Not studied

Lin et al. [113]	*Uncaria hirsuta* 95% ethanol and water extracts of stems with hooks 80 mg/kg/day p.o. for 4 or 8 weeks Male BALB/c mice, 8 week-old	D-Galactose-induced neurotoxicity (12 g in 100 mL of normal saline) 0.1 mL per 10 g of weight, s.c. once a day, for 4 or 8 weeks Plasma 4 weeks: MDA ↑ Plasma 8 weeks: MDA ↑ Brain 8 weeks: MDA ↑	Ethanol extract: Plasma MDA: 4 weeks (++), 8 weeks (+++) Brain MDA: 8 weeks: (++) Water extract: Plasma MDA: 4 weeks (++); 8 weeks (+++) Brain MDA: 8 weeks (++)	Not studied

Mohale et al. [111]	*Nerium indicum* flowers ethyl acetate extract 200 and 400 mg/kg, once daily p.o. Male Sprague-Dawley rats, 200–250 g	Anxiety induced by 21-day isolation in dark room Blood: LPO ↑, SOD, CAT, GSH ↓ Brain: LPO ↑, SOD, CAT, GSH ↓	Blood: GSH (+ both doses), SOD (++ both doses), CAT (++ 200, +++ 400), LPO (+++ both doses); Brain: CAT (++) both doses, SOD and GSH (++ 200, +++ 400), LPO (+++ both doses)	Not studied

Wu et al. [112]	*Malva sylvestris* L. leaf and flower 95% methanol extract 250 mg/kg per day, i.g., for 7 days Adult SPF-grade mice	Lipopolysaccharide-induced depression 250 *μ*g/kg i.p., a day before treatment (prior to injection sulindac sulfide 3.75 or 7.5 mg/kg for >3 weeks) Cortex: astrogliosis, IL-1, IL-6, TNF-*α* ↑, number of survival neurons ↓ Hippocampus: astrogliosis, IL-1, IL-6, TNF-*α* ↑, number of survival neurons ↓	Cortex: number of survival neurons, astrogliosis, IL-1, IL-6, TNF-*α* (++) Hippocampus: number of survival neurons, astrogliosis, IL-1, IL-6, TNF-*α* (++)	Not studied

↓: a decrease vs. control; ↑: an increase vs. control; (+): a slight beneficial effect; (++): a distinct beneficial effect; (+++): a complete beneficial effect; (0): no beneficial effect.

**Table 12:** The protective effects of plant origin materials in the animal model of menopause.

Reference	Plant extract, dose, way and time of treatment, and animals	Inducing factor and negative effects	Protective effects of plant material	Effects of plant *per se*
Leung et al. [115]	*Camellia sinensis* O. Ktze (black tea) extract (QP-lack tea extract, Quality Phytochemicals LLC) 15 mg/kg/day, i.g., for 4 weeks, 3 months after ovariectomy Female Sprague-Dawley rats, 3 months, 200–230 g	Ovariectomy Body weight: ↑ Serum: 17*β*-estradiol, cGMP ↓, non-HDL-c, HDL-c, TC, TG ↑ Aorta: peNOS ↓, NOX2, NOX4 ↑ Endothelium of aorta: ROS ↑	Body weight: (0) Serum: TG ↓, 17*β*-estradiol, non-HDL-c (0), HDL-c, TC (++), cGMP (+++) Aorta: peNOS, NOX2, NOX4 (+++) Endothelium of aorta: ROS (+++)	Not studied

Galanis et al. [114]	*Glycyrrhiza glabra* root methanol extract, in the form of drinking water at such a volume so as to obtain the upper threshold of glycyrrhizin (the main active component) of 12.4 mg/kg/rat per day, a day after surgery, for 3 or 6 months Female intact mature Wistar rats, 10 months	Ovariectomy Total tibia bone mineral density: after 3 and 6 months ↓ Proximal tibia bone mineral density: after both 3 and 6 months ↓ Uterine weight: after 6 months ↓	Total tibia bone mineral density: (+++ after 3 months, ++ after 6 months) Proximal tibia bone mineral density: after 3 months (+++), after 6 months (++) Uterine weight: (++)	Not studied

Hamm et al. [116]	*Humulus lupulus* L., hops flavonoid-rich extract (MetaGenics, Aliso Viejo, CA, USA) 400 mg/kg in food, daily, for 11 weeks Female C57BL/6 mice, 7 months	Ovariectomy Uterus weight ↓ Visceral adipose tissue weight ↑ Liver: TG ↑ Ileum: ALP ↑	Uterus weight (0) Visceral adipose tissue weight (+) Liver triglycerides (+++) Ileum: ALP (0)	Uterus weight ↓ Ileum: ALP ↑

↓: a decrease vs. control; ↑: an increase vs. control; (+): a slight beneficial effect; (++): a distinct beneficial effect; (+++): a complete beneficial effect; (0): no beneficial effect.

**Table 13:** The protective effects of plant origin materials in lung disorders.

Reference	Plant extract, dose, way and time of treatment, and animals	Inducing factor and negative effects	Protective effects of plant material	Effects of plant *per se*
Taguchi et al. [118]	Sakuranetin, a flavonoid obtained from methanol extract of aerial portions of *Baccharis retusa* by successive extraction and separation from CH_2_Cl_2_ phase Posttreatment -20 mg/kg *via* intranasal instillation on days 0, 7, 14, and 28 (the end of the experiment) C57BL6 male mice, 7-9-week-old, 25 g	Emphysema induced by elastase, 50 *μ*L (6.6 units/mg), an intranasal drop (0.667 IU) BALF: macrophages, lymphocytes, neutrophils, eosinophils, total cells ↑ Lung: NF-*κ*B, M-CSF, IL-1*β*, MCP-1, TNF-*α* ↑, expression of TIMP-1, MMP-9, MMP-12 ↑ Lung: collagen and elastic fiber deposition ↑	BALF: eosinophils (-), macrophages, lymphocytes, neutrophils, total cells (++) Lung: MCP-1 (+), M-CSF, IL-1*β*, TNF-*α*, NF-*κ*B (+++), expression of TIMP-1 ↑, expression of MMP-9, MMP-12 (++) Lung: collagen and elastic fiber deposition (++)	BALF: eosinophils ↑

Kaveh et al. [117]	*Portulaca oleracea* leaves, 70% ethanol extract 1, 2 and 4 mg/mL in drinking water for 21 days Male Wistar rats, about 220 g	Asthma rats sensitized on days 1, 2, and 3 by 1 mg/kg of ovalbumin (OVA)+100 mg Al(OH)_3_, i.p., and exposed to 1% OVA aerosol for 20 min on days 6, 9, 12, 15, 18, and 21 Serum: thiol, SOD, CAT ↓, NO_2_^−^, NO_3_^−^, MDA ↑ Blood: lymphocytes ↓, total WBC, eosinophils, neutrophils ↑	Serum: thiol and MDA (0 1, + 2, ++ 4), SOD (0 1, + 2, +++ 4), CAT (0 1, ++ 2 and 4), NO_2_^−^ (++ 1, +++ 2 and 4), NO_3_^−^ (++ all doses) Blood: neutrophils and lymphocytes (0 1, ++ 2, +++ 4), eosinophils (0 1, +++ 2 and 4), total WBC (+++ all doses)	Not studied

Wang et al. [119]	Salvia miltiorrhiza Bge. f. alba root aqueous extract 4.6 or 14 g/kg b.w. daily in the drinking water, 21 days after MCT Male Sprague-Dawley rats, 200-220 g	Pulmonary hypertension animal model caused by monocrotaline (MCT)—a single dose of 60 mg/kg b.w., s.c. Mean pulmonary artery pressure, right ventricular systolic pressure ↑ Plasma: NO, 6-Keto-PGF1*α* ↓, ET-1 and TXB_2_ ↑ Lung: TGF-*β*1 ↑	Mean pulmonary artery pressure and right ventricular systolic pressure (++ both doses) Plasma: 6-Keto-PGF1*α* and TXB_2_ (++ both doses), NO, ET-1 (++ 4.6, +++ 14) Lung: TGF-*β*1 (++ both doses)	Not studied

↓: a decrease vs. control; ↑: an increase vs. control; (+): a slight beneficial effect; (++): a distinct beneficial effect; (+++): a complete beneficial effect; (0): no beneficial effect; (-): intensification of the harmful effect.

**Table 14:** The protective effects of plant origin materials in lipid profile disturbances.

Reference	Plant extract, dose, way and time of treatment, and animals	Inducing factor and negative effects	Protective effects of plant material	Effects of plant *per se*
de Toledo Espindola et al. [121]	*Campomanesia adamantium* root aqueous extract 200 mg/kg b.w., p.o., 8 weeks after hyperlipidemia Wistar rats, 60 days, 140-150 g	Hyperlipidemia induced by high fructose diet for 16 weeks Body weight gain: ↑ Serum: TC, TG ↑	Body weight gain: (++ during the experiment, + in the end) Serum: TC, TG (+++)	Not studied

Veber et al. [34]	*Brassica oleracea* L. red cabbage aqueous extract 125 or 250 mg/kg, p.o., twice a day, for 3 days Male Wistar rats, 200-300 g	Hyperlipidemia induced by a single administration of Triton WR-1339 400 mg/kg, i.p. Serum: MDA, PC ↑ RBC: SOD ↑, CAT ↓ Liver: MDA, PC, SOD, GPx ↑, CAT ↓ Kidney: MDA, PC, GPx ↑, SOD, CAT ↓ Cerebral cortex: SOD ↓, MDA, PC ↑ Hippocampus: SOD, CAT ↓, GPx, MDA ↑	Serum: MDA (0 both doses), PC (0 125, +++ 250) RBC: CAT (0 125, +++ 250), SOD (+++ both doses) Liver: CAT (0 125, +++ 250), PC (+++ 125, ++ 250), MDA, SOD, GPx (+++ both doses) Kidney: MDA(0 both doses), GPx (0 125, +++ 250), PC, SOD, CAT (+++ both doses) Cerebral cortex: SOD (0 125, +++ 250), PC (++ 125, +++ 250), MDA (+++ both doses) Hippocampus: SOD (0 125, +++ 250),MDA (++ 125, +++ 250), CAT and GPx (+++ both doses)	Not studied

Khlifi et al. [120]	*Erica multiflora* L. leaf methanolic extract Pretreatment 150 or 250 mg/kg, i.g., 1 hour before Triton WR-1339 Male Wistar rats, 150-200 g	Triton WR-1339-induced hyperlipidemia, a single dose 300 mg/kg, i.p. Serum: HDL-c ↓, TC, TG, VLDL-c, LDL-c↑ Plasma: ALT, AST ↑ Liver: SOD, GPx, CAT ↓, MDA ↑ Liver: total DNA damage ↑	Serum: HDL-c (0 150, ++ 250), TG, VLDL-c, LDL-c (++ both doses), TC (++ 150, +++ 250) Plasma: ALT (++ 150, +++ 250), AST (+++ both doses) Liver: SOD (+ 150, ++ 250), MDA (++ both doses), GPx (+ 150, +++ 250), CAT (++ 150, ++ 250) Liver: total DNA damage (++ both doses)	250 mg/kg only studied: Serum: VLDL-c, TG ↑ Liver: SOD ↑

Onyenibe et al. [122]	*Monodora myristica* fruit aqueous extract 100 or 200 mg/kg b.w., p.o., five times a week for 8 weeks Male Wistar albino rats, 120-140 g	Hypercholesterolemia induced by cholesterol, p.o. 40 mg/kg/0.3 mL, five times a week for 8 weeks Serum: HDL-c ↓, LDL-c, TC, TG, AST, ALT ↑ Liver: SOD, CAT, GSH ↓, MDA ↑ Heart: SOD, CAT ↓, MDA ↑	Serum: LDL-c (0 100, + 200), AST, TC (++ both doses), TG, ALT (+++ both doses), HDL-c (↑ both doses) Liver: MDA (++ both doses), SOD, CAT, GSH (+++) both doses Heart: MDA (++ 100, +++ 200), SOD (+++) both doses, CAT (↑ both doses)	Not studied

↓: a decrease vs. control; ↑: an increase vs. control; (+): a slight beneficial effect; (++): a distinct beneficial effect; (+++): a complete beneficial effect; (0): no beneficial effect.

**Table 15:** The protective effects of plant origin materials in an ischaemia/reperfusion model.

Reference	Plant extract, dose, way and time of treatment, and animals	Inducing factor and negative effects	Protective effects of plant material	Effects of plant *per se*
Caskurlu et al. [124]	*Nigella Sativa* seeds oil Pretreatment, a single dose of 400 mg/kg p.o., 3 days before reperfusion Male Sprague-Dawley rats, 250-300 g, 10-12 weeks	Renal reperfusion injury 45 min occlusion of the bilateral renal pedicles + reperfusion (24 h) Kidney: tubulointerstitial damage ↑, glomerular injury score ↑, GPx, CAT ↓, MDA ↑	Kidney: tubulointerstitial damage (++), glomerular injury score (++), GPx, CAT, MDA (++)	Not studied

Sravanthi and Rao [126]	*Pentapetes phoenicea* methanolic extract Pretreatment, for 10 days 100, 200 or 400 mg/kg/day Male Wistar rats, 300–350 g	Global cerebral ischemia induced by temporary bilateral carotid artery occlusion (30 min) and subsequent 4 h reperfusion Brain: weight, water and protein content, infarct volume and LPO ↑, SOD, CAT, GSH, GPx, GR, GST ↓	Brain: weight, water and protein content, infarct volume, MDA, SOD, CAT, GSH, GPx, GR, GST (++ all doses) The greater the dose the better the effect	Not studied

Cakir et al. [123]	*Hypericum perforatum* 80% ethanol extract 50 mg/kg, i.p., at the beginning of ischemia Male Sprague-Dawley rats, 300–350 g	Right nephrectomy and subsequent ischaemia (45 min)/reperfusion (3 h) of the left kidney Serum: BUN and creatinine ↑ Kidney: CAT, SOD, GPx ↓, MDA ↑ Kidney: hydropic changes, tubular dilation, tubular desquamation congestion ↑	Serum: BUN and creatinine (-) Kidney: CAT, SOD, GPx and MDA (+++) Kidney: tubular desquamation congestion, hydropic changes, tubular dilation (++)	Not studied

Godinho et al. [125]	*Trichilia catigua* ethyl-acetate fraction of acetone-water (7 : 3) extract 200 mg/kg, 0.5 h before and 1 h after surgery (total dose 400 mg/kg), p.o. Male Wistar rats, 3-month-old	Cerebral ischaemia-reperfusion 4-VO model of transient global cerebral ischaemia, 15 min ischaemia Brain: GSH, GSH/GSSG, CAT, SOD ↓, GSSG, PCG, MDA ↑	Brain: CAT (0), GSH, GSSG, GSH/GSSG, SOD, PCG, MDA (+++)	Not studied

↓: a decrease vs. control; ↑: an increase vs. control; (++): a distinct beneficial effect; (+++): a complete beneficial effect; (0): no beneficial effect; (-): intensification of the harmful effect.

**Table 16:** The protective effects of plant origin materials in animal models of diabetes.

Reference	Plant extract, dose, way and time of treatment, and animals	Inducing factor and negative effects	Protective effects of plant material	Effects of plant *per se*
Ben Salem et al. [128]	*Cynara scolymus* leaves 75% ethanol extract 200 or 400 mg/kg b.w. daily, p.o., for 28 days Male Wistar rats, 10-12-week-old, 160–200 g	Alloxan monohydrate-induced diabetes, a single dose 150 mg/kg b.w., i.p. Body weight gain: ↓ Blood glucose: ↑ Serum: *α*-amylase, pancreatic lipase, ALT, AST, ALP, urea, CR ↑ Plasma: TG, TC, LDL-c ↑, HDL-c ↓ Blood: RBC, WBC, HGB, PLT ↓ Liver: SOD, CAT, GSH ↓, MDA, AOPP ↑ Pancreas: SOD, CAT, GSH ↓, MDA, AOPP ↑ Kidney: SOD, CAT, GSH ↓, MDA, AOPP ↑	Body weight gain: (+++ both doses) Blood glucose: (+++ 200, ++ 400) Serum: ALP (++ both doses), urea (++ 200, +++ 400), CR (+++ 200, ++ 400), ALT, AST, *α*-amylase, pancreatic lipase (+++ both doses) Plasma: TG, TC, LDL-c, HDL-c (+++ both doses) Blood: WBC (0 200, ++ 400), PLT (++ both doses), RBC and HGB (+++ both doses) Liver: MDA, SOD, CAT, AOPP (+++ both doses), GSH (+++ 200, ↑ 400) Pancreas: SOD (++ both doses), MDA, CAT (+++ 200, ++ 400), GSH, AOPP (+++ both doses) Kidney: SOD (+ 200, ++ 400), CAT (+++ 200, ++ 400), GSH, MDA, AOPP (+++ both doses)	Not studied

Dra et al. [127]	*Caralluma europaea* aerial part extract obtained by successive extraction with dichloromethane and methanol 250 or 500 mg/kg b.w., p.o., a single dose, followed for 10 h Swiss albino mice, 8 weeks	Alloxan monohydrate-induced diabetes type 1, a single dose of 200 mg/kg b.w., i.p. Blood: glucose ↑ Islets diameter: ↓	The lower dose: Blood glucose: after 2, 4, and 6 h (++), after8 h (+++) and after 10 h (↓); islet diameter (++) The higher dose: Blood glucose: after 2 (++), after 4 and 6 h (+++), after 8 and 10 h (↓)	Not studied

Silva et al. [59]	*Musa × paradisiaca* L. green banana pasta Commercial feed containing 25, 50, or 75% of green banana pasta, for 12 weeks Male Wistar rats, 6-8 weeks old, 180-300 g	Alloxan-induced diabetes, type 1, i.p., a single dose of 150 mg/kg Serum: glucose, ALT, AST, TC, TG, fructosamine ↑ Liver: total lipids, LPO and PC ↑ Kidney: PC ↑	Serum: AST (0 all doses), glucose, fructosamine (+ 25 and 50, +++ 75), TC (0 25, +++ 50, 0 75), ALT (0 25, +++ 50 and 75), TG (+++ all doses) Liver: total lipids (+ 25 and 50, +++ 75), LPO (0 25, +++ 50, ↓ 75), PC (0 25, ++ 50, +++ 75) Kidney: PC (0 25, +++ 50, ↓ 75)	Not studied

Du et al. [66]	Polysaccharide separated from *Lycium barbarum* 100, 250, or 500 mg/kg, p.o., for 4 weeks Male Sprague Dawley rats 8 weeks old, 180–220 g	High-fat diet for 8 weeks and then diabetes induced by streptozotocin injection, 30 mg/kg for a week Fasting blood glucose: ↑ Serum: insulin, SOD, GPx ↓, BUN, IL-2, IL-6, TNF-*α*, INF-*α*, MCP-1, ICAM-1 ↑ Urine: ALB ↑	Fasting blood glucose: (+ 100, ++ 250, and 500) Serum: INF-*α* (0 100 and 250, ++ 500), insulin, IL-2, ICAM-1 (0 100, + 250, ++ 500), SOD, TNF-*α*, BUN (0 100, + 250, +++ 500), GPx, IL-6 (0 100, +++ 250 and 500), MCP-1 (+ 250, +++ 250 and 500) Urine: ALB (+ 250, ++ 250 and 500)	Not studied

Wattanathorn et al. [42]	The combination of 50% hydroalcoholic extracts of *Mangifera indica* L. seeds and *Polygonum odoratum* L. aerial parts prepared at a ratio 1 : 5 2, 10, or 50 mg/kg b.w., daily, for 10 weeks Male Wistar rats, 180–220 g	Diabetes cataract and retinopathy induced by a single injection of streptozotocin 55 mg/kg b.w. Blood glucose after 5 and 10 weeks ↑ Grade of cataract severity ↑ Total retinal thickness ↓ Lens: SOD, CAT, GPx ↓, MDA, VEGF, ERK1/2, p38MAPK, AR ↑ Lens: opacity index ↑	Blood glucose after 5 weeks: (+ 2 and 50, ++ 10) Blood glucose after 10 weeks: (+ 2, 0 10, - 50) Total retinal thickness: (++ 2 and 10, 0 50) Grade of cataract severity: (++ all doses) Lens: SOD (++ 2, + 10, - 50), MDA (++ 2 and 10, 0 50), GPx (+ 2 and 50, ++ 10), CAT, AR, ERK1/2, p38MAPK (++ all doses), VEGF (+ 2, ++ 10, +++ 50) Lens: opacity index (++ all doses)	Not studied

Balbaa et al. [57]	*Nigella sativa* seed oil 2.0 mL/kg b.w., i.g., for 21 days after diabetes development Male Wistar rats, about 125 g	Type 2 diabetes induced by a high-fat diet for 2 weeks, followed by a single injection of streptozotocin 35 mg/kg, i.p. Serum: TNF-*α*, IL-6, AGE ↑ Brain: TBARS, XO, NO, TNF-*α*, IL-6, IL-1*β*, iNOS, AGE, AChE, A*β*-42, IDE ↑, GSH, GPx, GST, SOD, glucose ↓	Serum: TNF-*α*, IL-6 (+++), AGE ↓ Brain: AChE (↓), TBARS, XO, IL-6, IL-1*β* (++), NO, TNF-*α*, iNOS, GSH, GST, SOD, AGE, A*β*-42, IDE, glucose (+++), GPx (↑)	Serum: IL-6 and AGE ↓ Brain: AChE and A*β*-42 ↓, TBARS, NO, IDE, GPx, GST, SOD ↑

Fajri et al. [131]	*Equisetum arvense* aerial parts, methanolic extract 250 or 500 mg/kg b.w., daily p.o., for 45 days after diabetes inducing Male mice, 6-8 weeks	Streptozotocin-induced diabetes, 50 mg/kg b.w. i.p., for 5 days Sperm: viability, motility ↓, percentage of morphological abnormalities, DNA damage and nuclear immaturity ↑	The lower dose: viability, percentage of morphological abnormalities and DNA damage (++), motility and percentage of nuclear immaturity (+++) The higher dose: viability, motility (++), percentage of morphological abnormalities, DNA damage and nuclear immaturity (+++)	Not studied

Khanra et al. [129]	*Abroma augusta* L. leaf methanol extract 100 or 200 mg/kg, p.o., daily for 4 weeks Male Wistar rats, 2–3-month-old, about 180 g	Type 2 diabetes induced by a single injection of streptozotocin 65 mg/kg i.p., 15 min later nicotinamide 110 mg/kg, i. p. Fasting blood glucose: ↑ Serum: HDL-c, insulin ↓, TC, TG, HbA1c, ALT, AST, urea, LDH, CK, CRP ↑ Kidney: DNA fragmentation, ROS, TBARS, PC, IL-1*β*, IL-6, TNF-*α*, ↑, total coenzymes Q9 and Q10, GSH, CAT, SOD, GST, GPx, GR, G6PD, ATP, Bcl-2/Bax ↓ Kidney: expression of NF-*κ*B, PKC (*α*, *β*, *δ*, *ε*), caspase 3, caspase 9 ↑ Heart: ROS, TBARS, PC, IL-1*β*, IL-6, TNF-*α*, GPx, GR, DNA fragmentation ↑, total coenzymes Q9 and Q10, GSH, CAT, SOD, GST, G6PD, ATP, Bcl-2/Bax ↓ Heart: expression of NF-*κ*B, caspase 3, caspase 9, PKC (*α*, *β*, *δ*, *ε*) ↑	Fasting blood glucose: (++ both doses Serum: TC, TG, HbA1c, ALT, AST, urea, LDH, CK, CRP, insulin (++ both doses), HDL-c (++ 100, +++ 200) Kidney: ROS, TBARS, PC, total coenzyme Q9, SOD, IL-1*β*, IL-6, TNF-*α*, DNA fragmentation, ATP and Bcl-2/Bax (++ both doses), total coenzyme Q10, GSH, GST, GPx, GR, G6PD (++ 100, +++ 200), CAT (+++ both doses) Kidney: expression of NF-*κ*B, PKC (*α*, *β*, *δ*, *ε*), caspase 3, caspase 9 (++ both doses) Heart: ROS, TBARS, PC, GSH, CAT, SOD, IL-1*β*, IL-6, TNF-*α*, ATP, Bcl-2/Bax, DNA fragmentation (++ both doses), GST, G6PD (++ 100, +++ 200), total coenzymes Q9 and Q10 (+++ both doses), GPx, GR (↑ both doses) Heart: expression of PKC (*α*, *β*, *δ*, *ε*), caspase 3, caspase 9 (++ both doses), NF-*κ*B (+++ both doses)	Not studied

Omodanisi et al. [130]	*Moringa oleifera* leaf extract obtained by successive extraction with n-hexane and 80% methanol, 250 mg/kg b.w., p.o., for 6 weeks Male Wistar rats, 10 weeks	Streptozotocin-induced diabetes, a single dose of 55 mg/kg, i.p. Plasma: glucose ↑ Serum: TP, ALB ↓ Kidney: weight and relative weight ↑ Kidney: GPx, SOD slightly ↓, CAT ↓, TNF-*α* slightly↑, MDA, IL-6 ↑	Plasma: glucose (++) Serum: ALB (+), TP (+++) Kidney: weight and relative weight (++) Kidney: GPx (-), CAT (0), TNF-*α*, IL-6 (+), SOD (++), MDA (+++)	Plasma: glucose ↓ Serum: TP, ALB ↑ Kidney: SOD ↑, GPx, IL-6, TNF-*α* ↓

↓: a decrease vs. control; ↑: an increase vs. control; (+): a slight beneficial effect; (++): a distinct beneficial effect; (+++): a complete beneficial effect; (0): no beneficial effect; (-): intensification of the harmful effect.

**Table 17:** The protective effects of plant origin materials in animal models of obesity.

Reference	Plant extract, dose, way and time of treatment, and animals	Inducing factor and negative effects	Protective effects of plant material	Effects of plant *per se*
de Oliveira et al. [43]	*Euterpe oleracea* Mart. (açaí) seed hydroalcoholic extract 300 mg/kg/d, i.g., for 12 weeks Male mice C57BL/6, 4 weeks	Diet-induced obesity 60% fat diet for 12 weeks Epididymal and retroperitoneal fat mass ↑ Body weight gain↑ Liver/body weight: ↑ Glikaemia: ↑ Plasma: adiponectin ↓, leptin ↑ Serum: TC, TG, LDL, VLDL ↑ Liver: SOD, CAT, GPx ↓, cholesterol, TG, MDA, PC ↑ Liver expression of lipogenic proteins: pAMPK, pACC/ACC ↓, SREBP-1c, HMG-CoA-R ↑	Epididymal and retroperitoneal fat mass (++) Body weight gain (++) Liver/body weight: (+++) Glikaemia: (++) Plasma: leptin, adiponectin (+++) Serum: TC, TG, LDL, VLDL (++) Liver: cholesterol, TG (++), SOD, CAT, GPx, PC (+++), MDA (↓), CAT (↑) Liver expression of lipogenic proteins: pAMPK, SREBP-1c, HMG-CoA-R, pACC/ACC (+++)	Liver: TG ↓, SOD ↑

Budriesi et al. [133]	*Castanea sativa* Mill bark extract (ENC®, SilvaTeam S.p.a., San Michele Mondovì, Italy) 20 mg/kg, p.o., for 21 days Male Sprague-Dawley rats, 9-week-old, 270-300 g	High-fat diet for 10 weeks and then during extract administration Body weight gain: ↑ Serum: TC, LDL-c, TG ↑, HDL-c ↓ Plasma: IL-1*α*, IL-1*β*, IL-2, IL-5, IL-6, IL-7, IL-12p70, IL-17A, IL-18, TNF-*α* ↑, IL-4, IL-10, IL-13 ↓ Liver: SOD, GSSG-red, UDPGT ↓ Ileum: MDA ↑ Colon: MDA ↑	Body weight gain: (++) Serum: TG (++), TC, LDL-c, HDL-c (+++) Plasma: IL-1*β* (-), IL-5, IL-18 (0), IL-7, TNF-*α* (+), IL-1*α*, IL-2, IL-6, IL-12pro70, IL-4, IL-10 (++), IL-17A, IL-13 (+++) Liver: UDPGT (++), SOD, GSSG-red (↑) Ileum: MDA (+++) Colon: MDA (++)	Liver: SOD ↑ Ileum: MDA ↑

El Ayed et al. [132]	10% ethanol extract of *Vitis vinifera* grape seed (50%) and skin (50%) 500 mg/kg b.w., i.p., daily for 6 weeks Male Wistar rats, 210–230 g	High-fat diet (40% fat, 45% carbohydrate, 15% protein) for 6 weeks Body and lung weight: ↑ Lung lipid content: ↑ Plasma: adiponectin, HDL-c ↓, CRP, TG, TC, VLDL-c, LDL-c/HDL-c, LDL-c ↑ Lung: CAT, SOD, Zn, Mg ↓, MDA, lipase, Ca, Fe ↑	Body and lung weight: (+++) Lung lipid content: (+++) Plasma: CRP and adiponectin (++), TG, TC, VLDL-c, LDL-c, LDL-c/HDL-c, HDL-c (+++) Lung: MDA (++), CAT, SOD, lipase, Mg, Zn, Ca, Fe (+++)	Plasma: TG, TC, VLDL-c, LDL-c, HDL-c, LDL-c/HDL-c ↓ Lung: Zn ↑, MDA, Mg ↓

Qiu et al. [61]	Hedansanqi Tiaozhi Tang (a Chinese herbal prescription) contains four traditional Chinese herbal medicines: *Panax notoginseng* dry root, *Salvia miltiorrhiza Bunge* dry root, *Crataegus pinnatifida Bge.* fruit, *Nelumbo nucifera Gaertn* leaf 350, 700, 1400 mg/kg/day, i.g., once a day, for 4 weeks Male Sprague-Dawley rats, 180–190 g	Hyperlipidaemia induced by high-fat diet (2% cholesterol, 10% lard, 10% egg yolk, 0.5% bile sodium, 77.5% standard diet (*w*/*w*), for 4 weeks Body weight ↑ Serum: TC, TG, LDL-c, AST, ALT, MDA ↑, HDL-c, SOD, CAT, GPx, T-AOC↓ Liver: T-AOC, SOD, CAT and GPx ↓, TC, TG, MDA ↑ Liver: steatosis, lobular inflammation, hepatocellular ballooning ↑	Body weight: (+ 350, ++ 700 and 1400) Serum: LDL-c, TC, (+ 350, ++ 700 and 1400), CAT (+ 350, +++ 700 and 1400), TG, GPx (++ 350 and 700, +++ 1400), T-AOC, MDA, SOD (++ 350, +++ 700 and 1400), HDL-c, ALT, AST, (+++ all doses) Liver: MDA (+ 350, +++ 700 and 1400), TC, TG, GPx (++ 350 and 700, +++ 1400), T-AOC, CAT (++ 350, +++ 700 and 1400), SOD (+++ all doses) Liver: steatosis (+ all doses), lobular inflammation (++ all doses), hepatocellular ballooning (++ 350 and 700, +++ 1400)	4200 mg/kg studied: none

Sheng et al. [33]	*Morus alba* var. *multicaulis (Perrott.) Loud.* (mulberry) leaves 20% mulberry leaf powder addition to high fat diet, 13 weeks Male C57BL/6J mice, 4-week-old, 15-20 g	Obesity caused by high fat diet (60% calories from fat), provided before (around 8 weeks) and during mulberry administration Body weight, fat mass ↑ BAT: body weight ratio ↓ Fasting blood glucose and plasma insulin ↑ HOMA-IR ↑ Serum: AST, CR↑	Body weight: (++ beginning from 6^th^ week of treatment) Fat mass and BAT: body weight ratio (++), fasting blood glucose and plasma insulin (++), HOMA-IR (+++) Serum: AST, CR (+++)	Not studied

↓: a decrease vs. control; ↑: an increase vs. control; (+): a slight beneficial effect; (++): a distinct beneficial effect; (+++): a complete beneficial effect; (0): no beneficial effect; (-): intensification of the harmful effect.

**Table 18:** The protective effects of plant origin materials in animal models of gastric ulcers.

Reference	Plant preparation, dose, way, time of treatment, and animals	Inducing factor and negative effects	Protective effects of plant preparation	Effects of plant *per se*
Rtibi et al. [135]	*Ceratonia siliqua* L. pod aqueous extract Pretreatment, 500, 1000, and 2000 mg/kg, b.w., p.o., during 15 days Male Wistar rats, 220–250 g	Ethanol 4 g/kg, b.w., p.o., one dose Ulcer index ↑ Ulcer mucus volume ↓ Stomach mucosa: MDA, H_2_O_2_ ↑, -SH groups, SOD, CAT, GPx ↓	Ulcer index: (++ all doses, a decrease along with an increased dose) Ulcer mucus volume: (++ 500 and 1000, +++ 2000) Stomach mucosa: SOD, CAT (++ 500 and 1000, +++ 2000); MDA (++ 500 and 1000, +++ 2000); H_2_O_2_, -SH groups, GPx (0 500, ++ 1000, +++ 2000)	Not studied

Chanda et al. [45]	*Paederia foetida* L. leaf methanol extract 100, 200 mg/kg b.w./day, for 4 days Wistar rats, 150-180 g	Indomethacin-pylorus ligation induced ulcer Indomethacin 25 mg/kg, s.c., once daily for 4 days + surgical procedure on the 4^th^ day Gastric secretion: volume, acid output ↑, pH ↓ Ulcer index: ↑	Gastric secretion: volume and acid output (++ both doses), pH (++ 100, +++ 200) Ulcer index: (++ both doses)	Not studied

Sabiu et al. [136]	*Spondias mombin* and *Ficus exasperata* leaf aqueous extracts Pretreatment 100 or 200 mg/kg b.w., p.o., for 21 days Wistar rats, about 180 g	Indomethacin-induced gastric ulceration, a single dose of 30 mg/kg b.w., p.o. Ulcer index: ↑ Gastric secretion: acid output ↑, pH ↓ Stomach: MDA ↑, SOD ↓ Gastric juice: pepsin activity ↑, mucin content ↓	Ulcer index: (++ both doses of both extracts) % ulcer inhibition: (++ both doses of both extracts) Gastric secretion: acid output and pH (++ both doses of both extracts) *Ficus exasperata* Stomach: MDA, SOD (++ both doses) Gastric juice: pepsin, mucin (++ both doses) *Spondias mombin* Stomach: MDA, SOD (++ both doses) Gastric juice: pepsin, mucin (++ both doses) The higher doses showed better beneficial effects	200 mg/kg b.w. only studied, slight effects Gastric secretion: acid output ↑, pH ↓ Gastric juice: mucin content ↓

Sattar et al. [137]	*Myristica fragrans* seeds 70% ethanol extract 200 mg/kg, an hour before ethanol, once a day for 15 days Wistar rats, 150-300 g	90% ethanol-induced gastric ulcers 5 mL/kg for 15 days, once a day Gastric contents: pH ↓, total acidity ↑; average number of ulcers per animal ↑ Ulcer index: ↑	Gastric contents: pH ↑, total acidity (++) Average number of ulcers per animal (++) Ulcer index: (++) % ulcer inhibition: (++)	Not studied

Raeesi et al. [134]	*Biebersteinia multifida* root 70% methanol extract Pretreatment 150 or 300 mg/kg, p.o., 1 h before ulcer induction Male Wistar rats, 200-250 g	75% ethanol-induced gastric ulcer 4 mL/kg, p.o. Ulcer area/stomach ↑; Number of ulcers/stomach ↑; Gastric mucosa: T-AOC ↓	Ulcer area/stomach: (+++ both doses) Number of ulcers/stomach: (++ both doses) Gastric mucosa: T-AOC (↑ both doses)	Not studied

↓: a decrease vs. control; ↑: an increase vs. control; (++): a distinct beneficial effect; (+++): a complete beneficial effect.

**Table 19:** The protective effects of plant origin materials in animal models of various diseases.

Reference	Plant extract, dose, way and time of treatment, and animals	Disorder, inducing factor, and negative effects	Protective effects of plant material	Effects of plant *per se*
Benhelima et al. [139]	*Nigella sativa* L. seeds essential oil, 5 mL/kg b.w. from 1^st^ to 28^th^ or from 15^th^ to 28th days, p.o. Male Wistar rats, 120-130 g	Lithiasis induced by 0.75% ethylene glycol (EG) and 1.0% ammonium chloride in drinking water for 28 days, first 3 days both, then only EG, for 25 days Body weight: ↓ Urine: Mg, Ca ↓, oxalate, UA, phosphate ↑ Serum: CR, UA, BUN ↑ Kidney: oxalate, Ca, phosphate ↑	From 1^st^ to 28^th^ Body weight: (++) Urine: Ca, Mg, oxalate, UA, phosphate (++) Serum: CR, UA, BUN (++) Kidney: oxalate, Ca, phosphate (++) From 15^st^ to 28^th^ Body weight: (++) Urine: Ca, Mg, oxalate, UA, phosphate (++) Serum: CR (+), UA, BUN (++) Kidney: oxalate, Ca, phosphate (++) The latter treatment showed worse effects	Not studied

Chang et al. [140]	*Lycium barbarum* fruit water extracts Blended: particles 3.58 ± 3.8 *μ*m Submicron: particles 100 ± 70 nm Dietary pretreatment: 250 mg/kg for 54 days, p.o. Male Sprague-Dawley rats, 8–10 week, 200-300 g	Retinal degeneration induced by white cool light, the intensity 1,400–1,500 lux for 2 days, 12 h light/12 h dark cycle Retinal thickness: ↓ Retina: GSSG+GSH ↓, MDA↑	Submicron extract: retinal thickness (++) Retina: GSSG+GSH ↑, MDA (+++) Blended extract: retinal thickness (+) Retina: GSSG+GSH (++), MDA (+++)	Not studied

Cojocariu et al. [141]	*Chrysanthellum americanum* polyphenolic (butanol) fraction of 80% ethanolic extract 100 mg/kg b.w., i.g., 2 days during and 4 days after stress paradigm Female Wistar rats about 200 g	Irritable bowel syndrome animal model induced by multifactorial stress exposure paradigm for 7 days Temporal lobe: SOD and GPx ↓, MDA ↑	Temporal lobe: SOD and GPx, MDA (++)	None

Sdayria et al. [41]	*Euphorbia retusa* leaf methanol extract Pretreatment 200 mg/kg, p.o. Swiss albino mice, 20–30 g	Inflammation, induced by 1% carrageenan (in 0.9%NaCl) injection of 100 *μ*L into the subplantar region of the right hind paw Hind paw: MDA↑, SOD, CAT, GPx ↓ Liver: SOD, CAT, GPx ↓	Hind paw: MDA, CAT, GPx, SOD (++) Liver: SOD, CAT, GPx (++)	Not studied

Hatipoğlu et al. [142]	*Crataegus orientalis* M Bieber. fruit 70% ethanol extract 100 mg/kg/day, orogastrically, at placement of the ligature, for 11 days Male Wistar rats, aged 4 months, about 340 g	Periodontitis caused by submerging a 4/0 silk ligature in the sulcus of the mandibular right first molars of rats, kept subgingivally for a period of 11 days Serum: TOS, OSI ↑, TAS ↓ Interdental area between first and second molars: inflammatory cells, osteoclasts, osteoblasts ↑	Serum: TOS, OSI, TAS (+++) Interdental area between first and second molars: osteoblasts (0), inflammatory cells and osteoclasts (++)	Not studied

Konda et al. [138]	*Azima tetracantha* root 95% ethanol extract Pretreatment 250 or 500 mg/kg, p.o., 60 min prior to glycerol Male Wistar albino rats, 150-250 g	Acute renal failure caused by hypertonic glycerol, a single dose of 8 mL/kg, i.m., into both hind limbs Serum: CR, BUN ↑, TP, ALB ↓ Urine output: ↓ Kidney: SOD, GSH, GR, GPx, CAT ↓	The lower dose: Serum: TP, CR, BUN, ALB (++) Urine output: (++) Kidney: GPx (0), CAT (+), SOD, GR (++), GSH (+++) The higher dose: Serum: TP, ALB (++), BUN, CR, (+++) Urine output: (+++) Kidney: SOD, GSH, GR, GPx, CAT (+++)	Only 500 mg/kg studied Urinary output: ↑ Kidney: SOD ↓

Panth et al. [36]	*Salicornia europaea* water extract 1400 mg/kg/day p.o., for 6 weeks Male rats: spontaneous hypertensive (SHR) rats and Sprague-Dawley, 6 weeks, about 200 g	Vascular dysfunction and hypertension, NaCl-induced, 800 mg/kg/day, p.o. for 6 weeks SD rats: systolic and diastolic pressure ↑ SHR rats: systolic and diastolic pressure ↑	SD rats: systolic pressure (+++), diastolic pressure (++) SHR rats: systolic pressure (++), diastolic pressure (+)	Not studied

El-Kashlan et al. [143]	*Phoenix dactylifera* L. date palm pollen (commercial product) 80% ethanol extract 150 mg/kg, p.o., every day for 56 days Male Wistar rats, about 250 g	L-thyroxine-induced hyperthyroidism 300 *μ*g/kg, i.p., every day for 56 days Weight: final body, testes, epididymis, prostate gland, seminal vesicle↓ Serum: fT3, fT4, E2 ↑, LH, FSH, T, TSH ↓ Testis: SDH, LDH, ALP, ACP, G6PD, CAT, SOD, GPx, GR, GSH ↓, MDA, NO, AST, ALT ↑ 6-N-propyl-2-thiouracil-induced hypothyroidism 10 mg/kg, i.p., every day for 56 days Weight: final body, testes, epididymis, prostate gland, seminal vesicle↓ Serum: fT3, fT4, LH, FSH, T ↓, E2, TSH ↑ Testis: SDH, LDH, ALP, ACP, G6PD, CAT, SOD, GPx, GR, GSH ↓, MDA, NO, AST, ALT ↑	Hyperthyroidism Weight: final body, testes, epididymis, prostate gland, seminal vesicle (++) Serum: fT3, TSH, E2 (++), fT4, LH, FSH, T (+++) Testis: ALP (++), SDH, LDH, ACP, G6PD, GR, GSH, MDA, NO, AST, ALT, GPx (+++), CAT, SOD ↑ Hypothyroidism Weight: final body, testes (0), epididymis, prostate gland, seminal vesicle (++) Serum: TSH, fT3, fT4 (0), E2 (++), LH, FSH, T (+++) Testis: ALP, NO (++), SDH, LDH, ACP, G6PD, GR, GSH, MDA, AST, ALT (+++), GPx, CAT, SOD ↑	Weight: prostate gland, epididymis, seminal vesicle ↑ Serum: fT3, fT4 ↓ LH, T, E2 ↑ Testis: SDH, LDH, GR, CAT, SOD, GPx, GSH ↑, MDA, NO ↓

You et al. [144]	Fermented papaya extracts (obtained by fermentation of papaya through *Aspergillus oryzae* for 3 months and then for 3 months using *Saccharomyces cerevisiae* yeasts) 30, 15 or 5 mL/kg. p.o., days 1–30 Female SPF Sprague-Dawley rats, 180–200 g, 7-8 weeks	Mammary gland hyperplasia induced by estrogen and progestin, 0.5 mg/kg of estradiol benzoate Days 1-25 and 4 mg/kg of progestin days 26 – 30, i.m. Serum: E2, progesterone, LH, FSH, MDA, AST, total bilirubin ↑; GPx, SOD, ALT ↓ Liver: MDA ↑; GPx, SOD ↓ Mammary gland: GPx, SOD ↓, hyperplasia, MDA ↑	Serum: MDA (0 5, ++ 15 and 30), progesterone (+ 5 and 15, ++ 30), total bilirubin (0 5, ++ 5, +++ 30), E2 (++ all doses), LH (+ 5, ++ 5, +++ 30), FSH, ALT, AST (++ 5 and 15, +++ 30); GPx and SOD (0 5, +++ 15 and 30) Liver: MDA (0 5, ++ 15 and 30), GPx, SOD (0 5, ++ 15, +++ 30) Mammary gland: hyperplasia (0 5, + 15, ++ 30), MDA, GPx (0 5, ++ 15 and 30), SOD (0 5, +++ 15 and 30)	Only 30 mL/kg studied Serum: SOD ↑ Liver: SOD ↑

↓: a decrease vs. control; ↑: an increase vs. control; (+): a slight beneficial effect; (++): a distinct beneficial effect; (+++): a complete beneficial effect; (0): no beneficial effect.

**Table 20:** The protective effects of plant preparations against radiation.

Reference	Plant preparation, dose, way and time of treatment, animals	The dose and negative effects	Protective effects of plant preparation	Effects of plant *per se*
Dong et al. [70]	*Spatholobus suberectus* Dunn (Ji-Xue-Teng Beijing Lvye Pharmaceutical Co. Ltd., China) 75% ethanol extract 40 g/kg, p.o., for 21 consecutive days after irradiation Chinese Kun Ming (KM) mice, 6–8 week aged	^60^Co *γ*-radiation, a dose of 6 Gy Body weight: ↓ Blood: WBC, RBC, PLT, HGB ↓ Bone marrow tissue: Bcl-2 expression, phosphorylation of JAK2 and STAT5a ↓ Liver: ROS, MDA↑, SOD, GPx ↓ Femur: bone marrow cells ↓	Body weight: (+++) Blood: WBC, PLT, HGB (++), RBC (+++) Bone marrow tissue: phosphorylation of JAK2 (++), phosphorylation of STAT5a and Bcl-2 expression (+++) Liver: ROS, MDA (++), SOD, GPx (+++) Femur: bone marrow cells (++)	Not studied

Jeena et al. [145]	*Zingiber officinale* R (ginger) essential oil (Kancore Ingredients Limited, Angamali, Kerala, India) 100 or 500 mg/kg b.w. p.o., 5-day pretreatment and then for 14 days Male Balb/C mice, 6-8 week	*γ*-Radiation, 6 Gy, whole body exposure Blood: WBC, HGB ↓ Intestinal mucosa: SOD, CAT, GPx, GSH ↓	Blood: WBC (++), HGB (+++) Intestinal mucosa: SOD CAT, GPx and GSH (++ 100, +++ 500)	Not studied

Khattab et al. [146]	*Borago officinalis* L. seeds oil Posttreatment: 50 mg/kg b.w./day, p.o., 3 hours after irradiation and for 2 weeks Pretreatment: 50 mg/kg b.w./day, p.o., one week before irradiation and for 2 weeks Male albino rats, 160-180 g	*γ*-Radiation a single sublethal dose of 6.5 Gy of whole body Serum: GSH, HDL-c ↓, AST, ALT, GGT, MDA, TG, TC, LDL-c ↑ Liver: GSH ↓, MDA ↑	Posttreatment: Serum: AST, ALT (++), GGT, MDA, TG, TC, GSH, LDL-c, HDL-c (+++) Liver: GSH (++), MDA (+++) Pretreatment: Serum: AST, ALT (++), GGT, MDA, TG, TC, GSH, LDL-c, HDL-c (+++) Liver: GSH (++), MDA (+++)	None

Wang et al. [147]	*Rubus idaeus* red raspberry ethanolic extract 750 *μ*g/mL applied on the dorsal region of the nude mice 1 day before UVB and then for 5 days Female nude mice (ICR-Foxn/nu strain)	UVB 30 mJ/cm^2^ in the dorsal region, once a day for 5 days Total dose of 150 mJ/cm^2^ per mouse Skin epidermal thickness: ↑ Transepidermal water loss ↑ Erythema ↑ Skin: COX-2, DNA oxidation and protein carbonylation ↑	Skin epidermal thickness: (+++) Transepidermal water loss (++) Erythema (++) Skin: COX-2, DNA oxidation (++), protein carbonylation (+++)	Not studied

↓: a decrease vs. control; ↑: an increase vs. control; (++): a distinct beneficial effect; (+++): a complete beneficial effect.

## Abbreviations

AA: Ascorbic acid
A*β*: *β*-Amyloid peptide
ABCG: ATP-biding cassette, subfamily G transporters
ACC: Acetyl-CoA carboxylase
AChE: Acetylcholine esterase
ACP: Acid phosphatase
ADA: Adenosine deaminase activity
AGE: Advanced glycation end product
AGGRECAN: Cartilage-specific proteoglycan core protein
ALB: Albumin
AlCl_3_: Aluminium chloride
ALP: Alkaline phosphatase
ALT: Alanine aminotransferase
AOPP: Advanced oxidation protein products
AR: Aldose reductase
ARE: Antioxidant-responsive element
AST: Aspartate aminotransferase
ATP: Adenosine triphosphate
BALF: Bronchoalveolar lavage fluid
BAP: Biological antioxidant power
BAT: Brown adipose tissue
Bcl-2: B-cell lymphoma 2
BMP2: Bone morphogenetic protein 2
BUN: Blood urea nitrogen
b.w.: Body weight
Ca: Calcium
Ca^2+^ATPase: Calcium-activated adenosine 5′-triphosphatase
CAT: Catalase
CCl_4_: Tetrachlorometan (carbon tetrachloride)
Cd: Cadmium
Cd36: Cluster of differentiation 36
cGMP: Cyclic guanosine-3′,5′-monophosphate
ChAT: Choline acetyltransferase
CHOP: C/EBP homologous protein
CK-MB: Creatinine kinase-MB
COL2: Collagen type-II
COL10: Collagen type 10
COMP: Cartilage oligomeric matrix protein
COX-2: Cyclooxygenase-2
CR: Creatinine
CRP: C-reactive protein
CS: Citrate synthase
cTnI: Cardiac troponin I
CTX-II: C-telopeptide of type II collagen
Cu: Copper
CYP2E1: Cytochrome P450-2E1
DBP: Diastolic blood pressure
DC: Diene conjugate
DHEAs: Dehydroepiandrosterone sulfate
DNA: Deoxyribonucleic acid
E2: Estradiol
eNOS: Endothelial nitric oxide synthase
ERK1/2: Extracellular signal-regulated kinase 1 and 2
ET-1: Endothelin-1
Fatp4: Fatty acid transport protein 4
Fe: Iron
FFA: Free fatty acids
FRAP: Ferric reducing ability of plasma
FSH: Follicle-stimulating hormone
fT3: Free T3
fT4: Free T4
*γ*-GCS: *γ*-Glutamyl cysteine synthetase
GGT: *γ*-Glutamyl transferase
GM-CSF: Granulocyte-macrophage colony-stimulating factor
G6PD: Glucose-6-phosphate dehydrogenase
GPx: Glutathione peroxidase
GR: Glutathione reductase
GRP78: 78 kDa glucose-regulated protein
GSH: Reduced glutathione
GSSG: The oxidized form of glutathione
GSSG-red: Oxidized glutathione reductase
GSSP: Glutationylated proteins
GST: Glutathione-S-transferase
HA: Hyaluronidase
HbA_1_c: Glycated hemoglobin
HCT: Haematocrit
HDL: High-density lipoprotein
HDL-c: High-density lipoprotein cholesterol
Hg: Mercury
HGB: Haemoglobin
HMG-CoA-R: 3-Hydroxy-3-methylglutaryl CoA reductase
HNE: 4-Hydroxy-2-nonenal
HO-1: Heme oxygenase 1
H_2_O_2_: Hydrogen peroxide
HOMA-IR: Homeostasis model assessment-insulin resistance
ICAM-1: Intercellular adhesion molecule
i.c.v.: Intracerebroventricular
IDE: Insulin degradation enzyme
IFN-*α*: Interferon-*α*
IFN-*γ*: Interferon-*γ*
i.g.: Intragastrically
IgG: Immunoglobulina G
IgM: Immunoglobulina M
IL: Interleukin
i.m.: Intramuscularly
iNOS: Inducible nitric oxide synthase
i.p.: Intraperitoneally
i.v.: Intravenously
JAK: Janus kinase
K: Potassium
KEAP1: Kelch-like ECH-associated protein 1
6-Keto-PGF1*α*: 6-Keto-prostaglandin F1 alpha
LDH: Lactate dehydrogenase
LDL: Low-density lipoprotein
LDL-c: Low-density lipoprotein cholesterol
LH: Luteinizing hormone
Lpl: Lipoprotein lipase
LPO: Lipid peroxidation
LPS: Lipopolysaccharide
MAO-A: Monoamine oxidase type B
MAO-B: Monoamine oxidase type B
MCP-1: Monocyte chemoattractant protein 1
M-CSF: Macrophage colony-stimulating factor
MDA: Malondialdehyde
MDSCs: Myeloid suppressor cells
Mg: Magnesium
Mg^2+^ATPase: Magnesium-activated adenosine 5′-triphosphatase
MIP: Macrophage inflammatory protein
MMP-1: Matrix metalloproteinase-1, interstitial collagenase
MMP-3: Matrix metalloproteinase-3, stromelysin-1
MMP-13: Matrix metalloproteinase-13, collagenase 3
MMP: Matrix metalloproteinase
MPO: Myeloperoxidase
mRNA: Messenger ribonucleic acid
MT: Metallothionein
MTH 1: A gene encoding 8-oxo-7,8-dihydrodeoxyguanosine triphosphatase
Na: Sodium
NADH: Nicotinamide adenine dinucleotide reduced form
Na^+^/K^+^ATPase: Sodium- and potassium-activated adenosine 5′-triphosphatase
NASH: Nonalcoholic steatohepatitis
NF-*κ*B: Nuclear factor-kappa B
NO: Nitric oxide
NO_3_: Nitrate
NO_2_: Nitrite
NOX: NADPH oxidase
NP-SH: Nonprotein sulfhydryl groups
NQO1: NAD(P)H:quinone oxidoreductase 1
Nrf2: Nuclear erythroid 2-related factor 2
8-OHdG: 8-Hydroxy-2′deoxyguanosine
OSI: Oxidative stress index
P: Phosphorus
P53: p53 protein
pACC: Phosphorylated acetyl-CoA carboxylase
pAMPK: Phosphorilated adenosine-monophosphate-activated protein kinase
Pb: Lead
PC: Protein carbonyls
PCG: Protein carbonyl groups
PCNA: Proliferating cell nuclear antigen
peNOS: Phosphorylated eNOS
PGE_2_: Prostaglandin E_2_
PGI2: Prostacyclin
PHGPx: Phospholipid hydroxyperoxide GPx
PKC: Protein kinase C
PLT: Platelets
P38 MAPK: p38 mitogen-activated protein kinase
pNF-*κ*B: Phospho-NF-*κ*B
p.o.: Orally
QR: Quinone reductase
RANTES: Regulated on Activation, Normal T-cell Expressed and Secreted
RBC: Red blood cells
ROS: Reactive oxygen species
SBP: Systolic blood pressure
s.c.: Subcutaneously
SDH: Sorbitol dehydrogenase
SH: Thiol groups
SHBG: Sex hormone-binding globulin
SOD: Superoxide dismutase
SPF: Specific pathogen free
Srebf1: Sterol regulatory element-binding transcription factor 1
SREBP-1c: Sterol-regulatory-element binding protein-1c
STAT: Signal transducer and activator of transcription
T: Testosterone
T-AOC: Total antioxidant capacity
TAS: Total antioxidant status
TBARS: Thiobarbituric acid reactive substances
TC: Total cholesterol
TG: Triglycerides
TGF-*β*: Transforming growth factor beta
tHcy: Total homocysteine
TIMP: Tissue inhibitor of metalloproteinases
TLC: Total leukocytic count
TLR: Tall-like receptor
TNF-*α*: Tumor necrosis factor alpha
TOS: Total oxidant status
TP: Total protein
TSH: Thyroid-stimulating hormone
TXA_2_: Thromboxane A2
TXB_2_: Thromboxane B2
UA: Uric acid
UDPGT: UDP-glucuronosyl transferase
VEGF: Vascular endothelial growth factor
VLDL: Very low-density lipoprotein
VLDL-c: Very low-density lipoprotein cholesterol
WBC: White blood cells
XO: Xanthine oxidase
Zn: Zinc.
